# Mitochondrial Imbalance in Down Syndrome: A Driver of Accelerated Brain Aging?

**DOI:** 10.14336/AD.2025.0189

**Published:** 2025-04-06

**Authors:** Xinxin Zuo

**Affiliations:** Department of Neurosciences, University of California San Diego, La Jolla, CA 92093, USA.

**Keywords:** aging, Down syndrome, oxidative stress, cell senescence, mitochondrial-targeted therapies

## Abstract

Down syndrome (DS), caused by trisomy of chromosome 21 (HSA21), is a complex condition associated with neurodevelopmental impairments and accelerated brain aging, often culminating in early-onset Alzheimer’s disease (AD). Central to this accelerated aging is mitochondrial imbalance, characterized by disrupted energy metabolism, increased oxidative stress, impaired dynamics, and defective quality control mechanisms like mitophagy. These abnormalities exacerbate neuronal vulnerability, driving cognitive decline and neurodegeneration. This review examines the genetic and biochemical underpinnings of mitochondrial dysfunction in DS, with a focus on the role of HSA21-encoded genes. We also highlight how mitochondrial dysfunction, amplified by oxidative stress and HSA21 gene dosage effects, converges with cellular senescence and neuroinflammation to accelerate Alzheimer-like pathology and brain aging in DS. Finally, we discuss emerging therapeutic strategies targeting mitochondrial pathways, which hold promise for mitigating neurodegenerative phenotypes and improving outcomes in DS.

## 1. Introduction

Mitochondria, the powerhouses of the cell, produce adenosine triphosphate (ATP) through oxidative phosphorylation (OXPHOS) and are central to energy metabolism [1]. They also regulate calcium homeostasis, oxidative stress, and programmed cell death, making them indispensable for cellular function and survival. Mitochondrial homeostasis is crucial for cellular integrity but deteriorates with age, contributing to cellular senescence and age-related disease onset. Evidence implicates mitochondrial dysfunction as a fundamental driver of neurodegeneration in Alzheimer's disease (AD), Parkinson’s disease, and Huntington’s disease [2]. These conditions share a common pathophysiology characterized by impaired ATP production, dysregulated mitochondrial dynamics, and excessive oxidative stress, leading to neuronal degeneration and cognitive decline. Mitochondrial dysfunction is a primary driver of neurodegenerative processes rather than a consequence of aging [3]. Within the brain, which has exceptionally high energy demands, mitochondrial deficits create a vicious cycle of metabolic insufficiency, reactive oxygen species (ROS) accumulation, and neuronal damage, accelerating neurodegeneration and cognitive impairment. Growing recognition of mitochondrial dysfunction as a key contributor to brain aging has spurred interest in targeted therapies to restore mitochondrial function and slow disease progression.

Down syndrome (DS), the most common genetic cause of intellectual disability, arises from complete or partial trisomy of chromosome 21 (HSA21; T21), affecting approximately 1 in 700 live births [4]. While DS is primarily classified as a neurodevelopmental disorder, it has a distinct neurodegenerative component. Individuals with DS have smaller total brain volumes, reduced cortical surface area, impaired neurogenesis, and neural progenitor cell (NPC) proliferation and differentiation deficits detectable in the fetal stage [5, 6], which contribute to characteristic widespread brain hypotrophy and cognitive impairments. Individuals with DS also display accelerated brain aging, with hallmark AD-like neuropathology (e.g., amyloid plaques, neurofibrillary tangles [NFTs]) emerging by the fourth decade of life. Dementia symptoms typically manifest in the fifth decade, underscoring the early and aggressive nature of neurodegeneration [7]. Given these pronounced structural and molecular changes, DS is a valuable model for studying interactions between neurodevelopmental and neurodegenerative processes [7, 8].

Studies have identified mitochondrial dysfunction as a central mechanism linking DS with premature brain aging and neurodegeneration [9]. In DS, mitochondria exhibit profound abnormalities as early as the fetal stage, including altered bioenergetics, disrupted dynamics, and excessive ROS production, leading to OXPHOS deficits, reduced ATP synthesis, and mitochondrial membrane potential (MMP) collapse across multiple tissues, including the brain, fibroblasts and erythrocytes. Overexpression of HSA21 genes disrupts gene regulation, leading to both upregulation and downregulation of targets and the shaping of DS phenotypic traits [10]. Overexpression of superoxide dismutase 1 (*SOD1*), amyloid precursor protein (*APP*), dual specificity tyrosine-phosphorylation regulated kinase 1A (*DYRK1A*), and regulator of calcineurin 1 (*RCAN1*) contributes to oxidative stress and mitochondrial dysregulation. Global transcriptional dysregulation of nuclear-encoded mitochondrial genes (NEMGs) further compromises mitochondrial biogenesis and OXPHOS, amplifying energy deficits and ROS accumulation [11]. DS neurons exhibit fragmented mitochondrial networks and reduced connectivity, mirroring mitochondrial defects in other neurodegenerative and progeroid syndromes [12]. Impaired mitophagy exacerbates the accumulation of dysfunctional mitochondria, fueling oxidative stress and neuronal damage. Collectively, these mitochondrial disturbances contribute to early-onset AD-like pathology and systemic cellular dysfunction in DS, establishing a mechanistic link between genetic alterations and accelerated neurodegeneration.

In this review, we first describe the hallmark features of brain aging and early-onset neurodegeneration in DS. We then explore the genetic and biochemical mechanisms underlying mitochondrial dysfunction, focusing on disrupted mitochondrial dynamics, impaired mitophagy, and oxidative stress, which we collectively refer to as “mitochondrial imbalance”. Finally, we discuss emerging therapeutic strategies for neurodegeneration aimed at restoring mitochondrial homeostasis in DS. By examining mitochondrial imbalance as a key factor in DS pathology, we integrate insights from mitochondrial biology, neurodegeneration, and aging to advance our understanding of how mitochondrial dysfunction drives cognitive decline and brain aging in DS.

## 2. Early-Onset Neurodegeneration and Accelerated Brain Aging in DS

Clinically, individuals with DS exhibit accelerated aging across multiple organ systems, including muscle hypotonia, osteoporosis, early-onset skin wrinkling, and sensory impairments (e.g., vision and hearing loss) [13]. DS is associated with an increased prevalence of endocrine dysfunction, including thyroid abnormalities, premature menopause, and diabetes, and immunodeficiency, characterized by reduced T and B lymphocyte counts and increased susceptibility to autoimmune disorders.

Aging is driven by interconnected cellular and molecular mechanisms—the “hallmarks of aging”. A pivotal 2023 review identified 12 key hallmarks that drive age-related decline, including genomic instability, telomere attrition, epigenetic alterations, proteostasis dysfunction, and mitochondrial impairment [14]. In DS, these hallmarks manifest with heightened severity, contributing to early-onset neurodegeneration and accelerated brain aging (Table 1).

At the genomic level, T21 is destabilizing, leading to excessive DNA damage and impaired repair mechanisms. Accelerated telomere attrition in DS suggests that cellular aging may begin as early as fetal development [15]. Widespread epigenetic abnormalities, including altered DNA methylation and histone modifications, exacerbate premature aging by dysregulating gene expression [16, 17]. Epigenetic clocks indicate that individuals with DS have an accelerated DNA methylation age, with blood and brain tissues appearing, on average, 6.6 years older than those of age-matched controls [18].

At the cellular level, proteostasis, the maintenance of protein homeostasis, is profoundly disrupted [19]. Aβ and hyperphosphorylated tau accumulation begins early in life, mirroring AD pathology while overwhelming already impaired macroautophagy and proteasomal degradation systems. Dysregulated nutrient-sensing pathways, particularly mTOR signaling cascade hyperactivity, exacerbate metabolic dysfunction and neuronal vulnerability [20]. Mitochondrial dysfunction, another key hallmark, manifests as deficient oxidative phosphorylation, increased oxidative stress, and unstable mitochondrial DNA [9]. These impairments are detectable as early as the fetal stage and persist throughout life, compounding energy deficits and oxidative damage that progressively impair neuronal function.

Systemically, DS is characterized by “inflammaging,” chronic low-grade inflammation and immune dysregulation that amplify neuroinflammation and neurodegeneration [13]. Alterations in both innate and adaptive immunity heighten susceptibility to infections and sustain neuroinflammation. Evidence also implicates gut microbiota imbalances in the link between systemic inflammation and brain aging. These mechanisms exacerbate intercellular signaling dysfunction and increase the vulnerability of DS brains to accelerate neurodegeneration.

**Table 1:** **Changes in Molecular Mechanisms in Normal vs**. DS Aging.

	Normal aging	DS aging
Genomic Instability	DNA damage accumulates gradually with age; mtDNA mutations and replication errors emerge later in life.	T21 leads to gene dosage effects (e.g., *APP*, *SOD1*, *DYRK1A*), causing increased ROS, replication stress, and impaired DNA repair.
Telomere Attrition	Telomere length shortens progressively over the lifespan, influenced by genetics and environmental factors.	Substantial telomere shortening occurs early, driven by oxidative stress and increased cell division rates.
Epigenetic Alterations	Gradual accumulation of epigenetic alterations, such as DNA methylation and histone modifications.	Abnormal DNA methylation and histone modifications occur as early as the prenatal period, disrupting gene expression related to brain development and function.
Loss of Proteostasis	Protein misfolding and aggregation emerge later, contributing to aging-related diseases such as AD.	Increased protein misfolding and aggregation, including early Aβ and tau accumulation, drive neurotoxicity.
Disabled Macroautophagy	Autophagy declines gradually with age, primarily associated with inflammation and metabolic dysregulation.	Impaired autophagy reduces clearance of damaged mitochondria and proteins, accelerating neurodegeneration.
Deregulated Nutrient-Sensing	Dysregulation occurs progressively with aging, leading to reduced metabolic efficiency and chronic inflammation.	mTOR hyperactivity and disrupted insulin signaling exacerbate metabolic imbalances and energy deficits in neurons.
Mitochondrial Dysfunction	Mitochondrial dysfunction develops gradually, contributing to energy deficits and oxidative stress.	Early mitochondrial dysfunction is characterized by reduced ATP production, elevated ROS, and disrupted dynamics.
Cellular Senescence	Senescent cells accumulate slowly, causing chronic inflammation and tissue dysfunction over time.	Premature senescence is induced by oxidative stress and telomere shortening; SASP amplifies inflammation and neurodegeneration.
Stem Cell Exhaustion	Stem cell function declines gradually, reducing tissue repair and regeneration capacity.	Neural and hematopoietic stem cell depletion occurs early, impairing regeneration and immune modulation.
Altered Intercellular Communication	Dysregulation emerges later, accompanied by chronic inflammation and metabolic disturbances.	Elevated inflammatory cytokines (e.g., IL-6) and hyperactive microglia/astrocytes exacerbate neuroinflammation.
Chronic Inflammation	Chronic inflammation develops gradually, often associated with metabolic syndrome and chronic diseases.	Persistent low-grade inflammation driven by ROS, SASP, and immune dysregulation amplifies tissue damage.
Dysbiosis	Microbial dysbiosis accumulates with age, influencing immune regulation and systemic inflammation.	Gut microbiota imbalance exacerbates systemic inflammation and affects brain-gut signaling and neuronal function.

DS, Down syndrome; mtDNA, mitochondrial DNA; T21, trisomy 21; APP, amyloid precursor protein; SOD1, superoxide dismutase 1; DYRK1A, dual-specificity tyrosine-(Y)-phosphorylation regulated kinase 1A; ROS, reactive oxygen species; AD, Alzheimer's disease; Aβ, amyloid-beta; mTOR, mechanistic target of rapamycin; SASP, senescence-associated secretory phenotype; IL-6, interleukin 6.

While multiple biomarkers and molecular signatures of aging are altered in DS, their precise relationships with aging remain unclear. Few studies have systematically explored these changes across different age cohorts or through longitudinal analyses; thus, it is difficult to distinguish aging-related alterations inherent to DS from those reflective of broader aging. Addressing these gaps is essential to our understanding of how DS intersects with aging biology. Although accelerated aging in DS affects multiple physiological systems (e.g., dermatological, sensory, endocrine, musculoskeletal), neurological decline has the most profound impact on quality of life [21]. Other progeroid syndromes such as Werner syndrome (WS) and Hutchinson-Gilford progeria syndrome (HGPS) also exhibit features of accelerated systemic aging, they differ markedly from DS in their impact on the brain. In WS, cognitive decline is uncommon and AD-like neuropathology is rarely observed, despite significant peripheral aging and shortened lifespan [21]. Similarly, HGPS patients display severe multisystem deterioration but typically retain normal cognitive function [21]. The strong association between DS and AD emphasizes the brain as a crucial site of premature aging. We focus on neurodegeneration and brain aging in DS, aiming to elucidate the mechanistic underpinnings and broader implications for aging-related diseases.

## 3. Mitochondrial Imbalance in the DS Brain

In the DS brain, mitochondrial dysfunction impairs bioenergetics, disrupts mitophagy, and causes excessive oxidative stress and abnormal mitochondrial dynamics, contributing to neuronal vulnerability and accelerated aging. These disruptions reveal a broader underlying theme, which we conceptualize as mitochondrial imbalance to capture the pervasive disequilibrium in mitochondrial homeostasis spanning molecular, structural, and systemic levels. This concept highlights the dynamic and interconnected nature of these disturbances, offering a broader perspective on their roles in premature aging and neural decline in DS. Next, we examine key dimensions of mitochondrial imbalance in detail, elucidate molecular mechanisms and explore implications for DS pathophysiology and therapeutic development.

### 3.1 Gene Dosage Imbalance: The Foundation of Mitochondrial Imbalance in DS

#### 3.1.1 Trisomy 21

DS, first documented by Dr. John Langdon Down in 1862, is the most prevalent chromosomal condition linked to intellectual disability [22]. The cause, inheritance of three (vs. two) copies of HSA21, was determined nearly a century later by Jérôme Lejeune and colleagues [23]. As the smallest human chromosome, HSA21 has been extensively sequenced, and a nearly complete, high-quality assembly of its long arm (21q) was published in 2000 [24]. Spanning approximately 33.6 Mb and constituting ~1% of the human genome, HSA21 harbors an estimated 233 protein-coding genes, 423 non-protein-coding genes, and 188 pseudogenes according to GENCODE/ENSEMBL annotations [11].

Most individuals with DS (95%) have a complete T21 (extra full copy in all cells). Less common variants (3%-4%) include mosaic DS, where a subset of cells carries T21 while others have a typical karyotype, and partial trisomy, where only part of HSA21 is triplicated. As DS primarily arises from this genetic imbalance, extensive research has focused on HSA21-encoded genes. *SOD1* was the first HSA21 gene studied in a transgenic mouse model of overexpression [25]. In 1990, Myriel Davisson at the Jackson Laboratory developed Ts65Dn, a viable mouse with partial trisomy 16 carrying a chromosomal segment homologous to a large portion of HSA21 (~132 genes) and exhibiting cognitive deficits and neurophysiological abnormalities [26]. Ts65Dn mice have since been widely used to study the molecular basis of DS.

Gene dosage imbalance is the key driver of the complex DS phenotype. While triplicated genes on HSA21 are generally upregulated, expression levels vary due to compensatory mechanisms, regulatory networks, and tissue-specific factors. In Ts65Dn mice, quantitative RT-PCR revealed that only ~37% of triplicated genes reached the expected 1.5-fold increase; ~45% were expressed below this level, and ~18% exceeded it [27, 28]. Regional differences were observed, with more pronounced gene overexpression in the cortex (~1.63-fold) than the cerebellum (~1.37-fold) and midbrain (~1.30-fold), reflecting varying molecular complexity [28]. Human transcriptome studies further confirmed that not all HSA21 genes follow the anticipated dosage effect. Among 255 genes analyzed, only 77 consistently showed increased expression, while others (e.g., mitochondrial ribosomal protein S6 [*MRPS6*], cystathionine-beta-synthase [*CBS*]) exhibited tissue- and species-specific variability. *MRPS6* was upregulated in the brain but downregulated elsewhere, whereas *CBS* showed opposing trends in humans and mouse models, suggesting species-specific regulatory differences [29]. Dosage-sensitive genes are enriched in some regions (e.g., chr21q22, chr21q21) indicating spatial heterogeneity. Trisomy-induced dysregulation also causes genome-wide regulatory disruptions. These findings underscore the complexity of gene dosage effects, revealing how trisomy affects gene expression in a context-dependent manner to shape brain development and function.

Gene dosage effects in T21 influence chromatin function through two primary mechanisms: (1) direct overexpression of HSA21 genes (coding and non-coding elements), and (2) dysregulation of gene expression across non-HSA21 chromosomes [30]. Chromatin alterations are not DS-exclusive and may arise in other aneuploidies or under cellular stress. Thus, DS is a valuable model for studying how aneuploidy disrupts chromatin dynamics and gene regulation and the interplay between genetic imbalance, chromatin architecture, and cellular function.

#### 3.1.2 HSA21-encoded Mitochondrial-Related Genes

Recent advancements have deepened our understanding of genotype-phenotype relationships in DS, specifically interactions between HSA21 gene overexpression and genome-wide dysregulation as key drivers of pathology. Several HSA21-encoded genes, including *APP*, *SOD1*, *RCAN1*, *DYRK1A*, *CBS*, nuclear receptor interacting protein 1 (*NRIP1*), and small ubiquitin-related modifier 3 (*SUMO3*), are linked to mitochondrial function, making mitochondrial dysfunction a central feature of DS.

**Figure 1.**
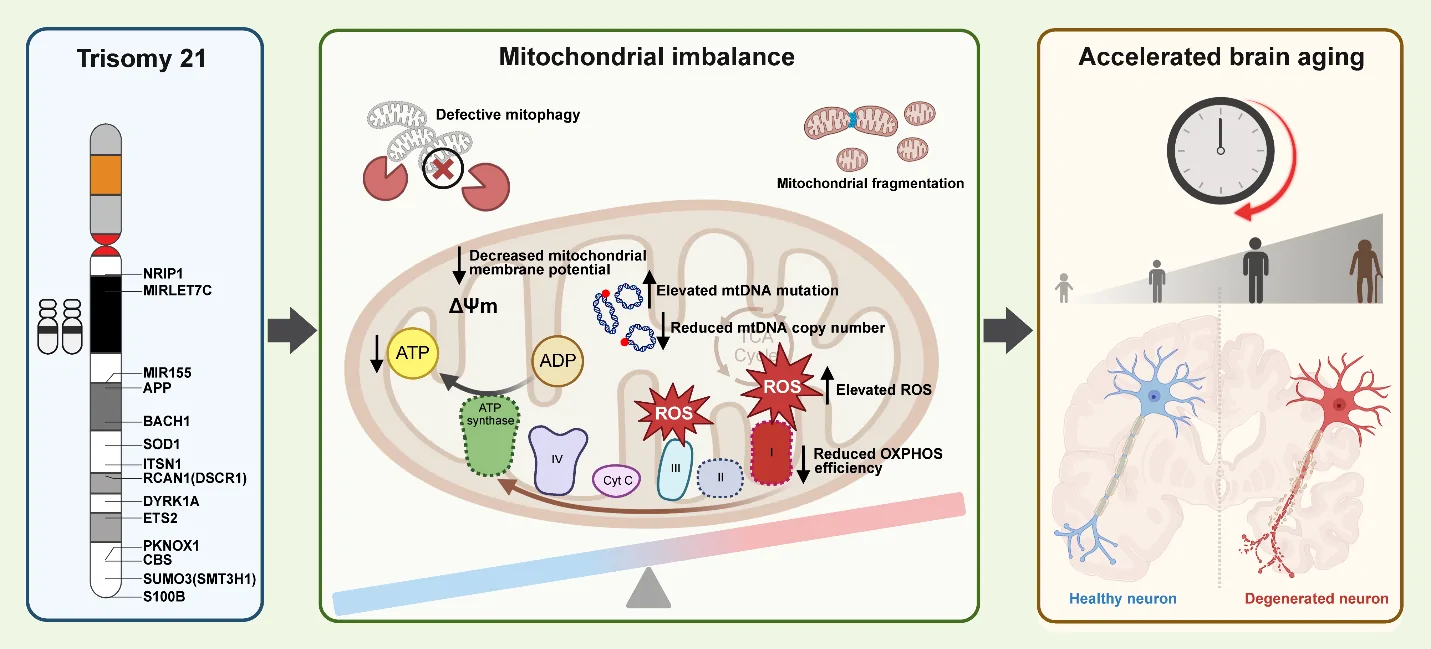
**HSA21 gene overexpression disrupts mitochondrial balance and accelerates brain aging in DS**. T21 leads to the overexpression of multiple HSA21-encoded genes involved in mitochondrial function, including *SOD1*, *APP*, *RCAN1*, *NRIP1*, *SUMO3*, *DYRK1A*, and *CBS*. These genes collectively affect redox balance, mitochondrial dynamics, and biogenesis. Gene dosage imbalance disrupts mitochondrial homeostasis by increasing reactive oxygen species (ROS), impairing mitophagy, and promoting mitochondrial fragmentation. Mitochondrial bioenergetic dysfunction is marked by reduced mitochondrial membrane potential (MMP, ΔΨm), elevated mtDNA mutations, reduced mtDNA copy number, and decreased OXPHOS efficiency. These alterations result in ATP depletion and contribute to neurodevelopmental deficits, neurodegeneration, and hallmark features of DS, including early-onset Alzheimer's disease-like pathology. Together, they establish a trajectory of mitochondrial vulnerability that underlies and accelerates brain aging in individuals with DS.

Impaired mitochondrial dynamics, structural abnormalities, and bioenergetic deficits, including OXPHOS deficiencies and reduced ATP production, contribute to hallmark DS traits such as accelerated aging and neurodegeneration. HSA21 mitochondrial gene overexpression disrupts cellular energy homeostasis, impairs neuronal development, and promotes neurodegeneration, emphasizing mitochondrial dysfunction as a key contributor to DS pathophysiology and linking gene dosage imbalance with complex phenotypic manifestations (Fig. 1). Overexpression of HSA21 mitochondrial-related genes has profound implications for mitochondrial function. *SOD1* is overexpressed in DS, leading to hydrogen peroxide (H_2_O_2_) accumulation [31]; this damages mitochondria, exacerbating oxidative stress and impairing mitochondrial dynamics if not decomposed by catalase (CAT) or glutathione peroxidase (GPx). Redox imbalance is pervasive in DS tissues, contributing to chronic oxidative stress. *APP* is another dosage-sensitive gene. Its overexpression leads to Aβ42 accumulation within the mitochondrial matrix, impairing electron transport, reducing OXPHOS efficiency, and triggering apoptosis [32, 33]. Subsequently, Aβ-mediated mitochondrial dysfunction amplifies oxidative stress and neuronal damage. *RCAN1* (*DSCR1*), a dosage-sensitive gene, maintains mitochondrial function and integrity [34]. Its overexpression disrupts mitochondrial permeability transition pore (mPTP) function, causing calcium overload, impaired Ca^2+^ retention, mitochondrial swelling, and outer membrane rupture [35]. DYRK1A contributes to mitochondrial dysfunction in DS by phosphorylating the import receptor TOM70 at Ser91, enhancing its interaction with the TOM core complex and the import of nuclear-encoded mitochondrial proteins essential for mitochondrial maintenance [36]. ETS2 exacerbates mitochondrial dysfunction by activating mitochondrial-dependent apoptosis. *ETS2* overexpression in DS neurons induces cytochrome c and apoptosis-inducing factor release, leading to caspase activation and neuronal death [37].

#### 3.1.3 Genome-Wide Dysregulation of Mitochondrial Function

T21 also leads to global transcriptional dysregulation, exacerbating mitochondrial dysfunction and impacting expression of more than 90 NEMGs in DS brain tissues [29]. These changes affect key processes such as OXPHOS, mitochondrial biogenesis, and stress responses. Upregulation of *MRPS6* in the brain indicates compensatory mechanisms for mitochondrial protein synthesis. Downregulation of *NDUFB8*, a key OXPHOS complex I gene, impairs energy production. *HSPA5*, a mitochondrial stress response gene, is consistently upregulated, while *DNAJB1*, a mitochondrial chaperone gene, is downregulated, contributing to protein-folding deficiencies. In summary, T21 both upregulates and downregulates mitochondrial pathways, with broad implications for brain development and neurodegeneration in DS. In DS-induced pluripotent stem cells (DS-iPSCs), mitochondrial dysfunction is linked to mitochondrial gene downregulation and widespread alterations in long noncoding RNA (lncRNA) expression. Functional enrichment analysis indicated that differentially expressed lncRNAs influence mitochondrial processes such as ATP synthesis and membrane organization, indicating potential regulation of DS-related mitochondrial dysfunction [38].

Upregulation of chromosome 21-derived microRNAs (miRNAs), including miR-155 and let-7c, further disrupts mitochondrial function by suppressing key NEMGs. When upregulated, miR-155 suppress mitochondrial transcription factor A (TFAM), a key regulator of mitochondrial DNA replication and transcription, disrupting mitochondrial biogenesis [39]. Increased let-7c expression targets solute carrier family 25 member 4 (SLC25A4/ANT1), a crucial regulator of ADP/ATP translocation, disrupting energy metabolism [40]. These imbalances highlight the extensive impact of gene dosage effects on mitochondrial dysfunction in DS.

### 3.2 Oxidative Stress and Redox Imbalance

ROS are generated during cellular metabolism and are essential for maintaining homeostasis and mediating cell signaling under normal conditions. In excess, they can induce oxidative stress, damaging lipids, proteins, and nucleic acids and compromising cellular integrity and function [41]. In DS, elevated oxidative biomarkers, including protein carbonylation, lipid peroxidation, and antioxidant system impairment, have been identified in the amniotic fluid of DS fetuses, underscoring mitochondrial dysfunction and oxidative stress as key contributors to DS pathogenesis [42].

#### 3.2.1 Excessive ROS Production in DS

ROS from endogenous and exogenous sources contribute to the cellular oxidative burden. Endogenously, mitochondria generate approximately 90% of total cellular ROS [7]. Other intracellular sources include NADPH oxidases (NOX) during immune responses and signal transduction, and peroxisomes during fatty acid oxidation [43, 44]. Mitochondrial ROS formation is a byproduct of OXPHOS, the central process driving ATP synthesis. OXPHOS relies on regulated NADH and FADH oxidation to drive proton transport across the mitochondrial inner membrane and establish an electrochemical gradient that powers ATP synthesis via F1-F0 ATPase (Complex V), catalyzing ADP phosphorylation to yield ATP [45]. OXPHOS includes the electron transport chain (ETC) and ATP synthase, both within the mitochondrial inner membrane. The ETC comprises Complexes I-IV (CI-CIV), coenzyme Q (Q), and cytochrome c. Electron leakage at CI and CIII produces superoxide anions, the predominant mitochondrial ROS.

The brain is highly susceptible to oxidative stress due to high oxygen consumption, abundant polyunsaturated fatty acids, and limited antioxidant defenses. This susceptibility is further heightened in DS due to overexpression of HSA21 genes such as *SOD1* and *APP*, which contribute to mitochondrial redox imbalance and oxidative damage [4, 46]. Triplication of other HSA21 genes, such as *BACH1* and *S100B*, also enhances oxidative stress in DS. In the brain, increased BACH1 expression and suppressed heme oxygenase-1 (HO-1) activation impair the antioxidant response by disrupting the Bach1/HO-1/BVR-A axis, leading to early oxidative stress [47]. S100B overexpression in DS NPCs amplifies ROS production, triggering oxidative stress and activating stress response pathways [48].

#### 3.2.2 Antioxidant Deficiencies and Systemic Vulnerability

In DS, antioxidant defenses cannot compensate for oxidative stress driven by T21-induced *SOD1* overexpression. Despite normal or slightly upregulated CAT and GPx levels in some tissues, these remain insufficient to neutralize excess H_2_O_2_ generated by SOD1 [49, 50], overwhelming mitochondrial redox regulation as mitochondria are both a primary source and target of ROS.

Redox homeostasis requires various antioxidant molecules, including GSH, amino acids (arginine, taurine, creatine), trace metals (selenium, zinc), and vitamins (E, C) [51]. In DS, disruptions of these antioxidant systems exacerbate oxidative stress. The GSH system is particularly affected, with fetal DS fibroblasts showing reduced levels of GSH and an increased oxidized-to-reduced glutathione ratio (GSSG/GSH) [52, 53]. The fetal DS brain exhibits decreased expression of peroxiredoxin 2, a crucial enzyme that protects lipids and proteins from oxidative damage [54]. Deficiencies in non-enzymatic antioxidants further compromise the redox balance. Both children and adults with DS often exhibit reduced blood or plasma vitamin E, vitamin C, selenium, and zinc levels [55-57]. Taurine, a key antioxidant amino acid, is significantly depleted in the frontal cortex of DS fetal brains [58]. These deficiencies weaken endogenous antioxidant defenses, increasing susceptibility to oxidative stress and contributing to systemic vulnerability.

#### 3.2.3 Oxidative Damage and Cellular Consequences

Excessive ROS production in DS drives widespread oxidative damage across cellular components, generating highly reactive byproducts such as 4-hydroxynonenal (HNE), which propagate free radical formation [59]. In the fetal brain, lipid peroxidation markers, including significantly elevated malondialdehyde levels, indicate early oxidative stress [49]. In DS neurons, increased intracellular ROS and lipid peroxidation precede neuronal apoptosis in neurodegeneration [60]. Oxidative protein damage disrupts receptors, hormone, and enzyme functions, impairing intracellular degradation and promoting dysfunctional protein accumulation. A redox proteomics study of the frontal cortex in individuals younger than 40 with DS revealed elevated protein carbonylation, particularly affecting cellular quality control proteins such as cathepsin D, V0-ATPase, GRP78, and GFAP [61]. Oxidative damage impairs proteasome function and autophagosome formation, accelerating the Aβ and hyperphosphorylated tau accumulation, a key hallmark of DS-associated neurodegeneration.

DS pathology is complicated by ROS-induced DNA damage, including base modifications, strand breaks, and DNA-protein crosslinks. Fibroblasts and lymphocytes from individuals with DS exhibit elevated oxidative DNA damage, defective base excision repair (BER) mechanisms, and heightened oxidative stress sensitivity [62, 63]. RNA oxidation, indicated by upregulated 8-hydroxyguanine (8-OH-G), is highly elevated in DS neurons, particularly in early life. Aβ deposition appears to temporally follow oxidative stress, suggesting a potential compensatory response to mitigate oxidative damage [64].

However, not all studies have consistently reported elevated oxidative stress markers in DS. While some indicators such as 3-nitrotyrosine and HNE are reliably increased, others like TBARS and 15-F(2t)-isoprostanes have yielded variable results [65]. In adults with DS, oxidative DNA damage is not always elevated, suggesting possible age-related adaptations [66]. These inconsistencies highlight the need for stratified analysis by age, tissue type, and oxidative marker when interpreting redox imbalance in DS.

### 3.3 Structural and Functional Imbalance in Mitochondrial Dynamics

Mitochondria are double-membraned organelles with a specialized structure crucial for bioenergetics and cellular signaling. The outer mitochondrial membrane (OMM) interfaces with the cytosol, facilitating interactions with other organelles (e.g., endoplasmic reticulum [ER], peroxisomes, endosomes). The tightly folded and selectively permeable inner mitochondrial membrane (IMM) forms cristae that embed electron transport chain (ETC) complexes, facilitating OXPHOS [67]. In DS cells, mitochondrial morphology is abnormal, characterized by fragmentation, swelling, and cristae disorganization that impair mitochondrial function [68].

#### 3.3.1 Disrupted Fission and Fusion

Mitochondrial dynamics encompass fission and fusion and are crucial for mitochondrial integrity and cellular homeostasis [69]. Fusion, regulated by GTPases such as optic atrophy 1 (OPA1) in the IMM and mitofusins (MFN1/2) in the OMM, enables damaged mitochondria to merge and recover. Fission, controlled by dynamin-related protein 1 (DRP1), isolates dysfunctional mitochondrial fragments for degradation. In DS, this balance is disrupted, leading to excessive mitochondrial fragmentation and impaired mitochondrial function.

Mitochondrial dysfunction in DS appears to begin early in development. In DS fetal fibroblasts, reduced PGC-1α expression is associated with lower OPA1 and MFN2 levels, fragmented mitochondria, intra-mitochondrial edema, and cristae abnormalities [70]. Hippocampal NPCs in Ts65Dn mice exhibit excessive mitochondrial fragmentation, driven by increased DRP1 and reduced MFN2 and OPA1 expression [71]. In DS neurons and astrocytes, fragmented mitochondrial networks are linked to oxidative stress, elevated mitochondrial ROS, and decreased MMP and ATP synthesis, exacerbating cellular dysfunction [12].

Regulator of calcineurin 1 (RCAN1) overexpression is a hallmark of gene dosage imbalance in DS and a key contributor. RCAN1 upregulation enhances mitochondrial ROS production, disrupts mitochondrial repair mechanisms, and promotes mPTP opening, leading to Ca^2+^ dysregulation, mitochondrial swelling, OMM rupture, and increased susceptibility to cell death [35]. RCAN1 interferes with DRP1 activity, amplifying mitochondrial fragmentation, bioenergetic deficits, and oxidative stress and contributing to age-related neurodegeneration, including AD [72].

#### 3.3.2 Impaired Mitophagy and Mitochondrial Quality Control

Mitophagy, the selective removal of damaged mitochondria, is essential for mitochondrial integrity and preventing apoptosis [20]. Dysfunctional mitochondria are encapsulated by mitophagosomes, transported to lysosomes, and degraded, ensuring appropriate turnover. Mitophagy is impaired in DS, leading to mitochondrial accumulation, increased oxidative stress, and progressive dysfunction. Several HSA21-encoded gene products have been implicated in mitophagy dysregulation. ETS2 overexpression in DS neurons disrupts mitochondrial integrity by promoting Bax upregulation, cytochrome c release, and apoptosis, suggesting failed mitophagy-mediated quality control [37]. Intersectin 1 (ITSN1), an endocytic scaffold protein, regulates mitophagy; its deficiency leads to mitochondrial accumulation and cytochrome c-mediated apoptosis, partly through impaired MEK/ERK signaling [73].

Autophagy, including mitophagy, is inhibited by mTOR signaling, a key cell growth and metabolism regulator. In DS cells, mTOR complex 1 (mTORC1) hyperactivation disrupts autophagic flux, impairing mitochondrial clearance. DS fibroblasts, particularly fetal-derived cells, exhibit persistent mTORC1 hyperactivation as early as gestational week 19, which continues postnatally [74]. This dysregulation hinders autophagy initiation, autophagosome formation, and mitophagy, exacerbating mitochondrial dysfunction. A key mitophagy component, the ULK1 complex, is suppressed by mTORC1 phosphorylation, preventing autophagy initiation. ULK1 phosphorylates FUNDC1, an OMM protein, at Ser-17, enhancing its binding to LC3 and facilitating recruitment of damaged mitochondria into autophagosomes [75]. In DS fibroblasts, mTORC1 hyperactivation suppresses ULK1 activity, impairing autophagy and mitochondrial turnover [20].

The PINK1/Parkin pathway, essential for mitophagy, is also disrupted in DS. Under mitochondrial stress, PINK1 accumulates on the OMM and recruits the E3 ubiquitin ligase Parkin, which ubiquitinates damaged mitochondria to mark them for degradation [76, 77]. In DS fibroblasts, impaired PINK1/Parkin signaling is characterized by reduced Parkin expression and delayed PINK1 activation, leading to mitochondrial depolarization, ROS accumulation, and defective mitophagy [20].

### 3.4 Genetic and Biochemical Instability of mtDNA

MtDNA is a 16.5-kb, double-stranded circular genome essential for cellular energy production. It encodes 13 polypeptides crucial for OXPHOS, two ribosomal RNAs (rRNAs), and 22 transfer RNAs (tRNAs) necessary for mitochondrial protein synthesis [78]. Each mitochondrion contains multiple mtDNA copies. However, the majority of mitochondrial proteins, including those involved in mtDNA replication, transcription, translation, and OXPHOS complex assembly, are nuclear-encoded, reflecting the intricate nuclear-mitochondrial interaction required to maintain mitochondrial function.

#### 3.4.1 mtDNA Damage and OXPHOS Deficiencies

MtDNA is particularly vulnerable to oxidative damage due to its proximity to ROS production sites and limited repair capacity. In DS, this vulnerability is exacerbated by systemic mitochondrial dysfunction, leading to cumulative mtDNA alterations—a process that mirrors the mitochondrial cascade hypothesis proposed in AD, wherein early mtDNA damage is thought to initiate and accelerate neurodegenerative progression [79].

In DS brains, mitochondrial CI subunits (24-kDa and 75-kDa) are significantly reduced in the temporal and occipital cortices and caudate nucleus, impairing energy metabolism and neuronal function [80]. In Ts65Dn mice, oxygen consumption, CI and CIV activity, ATP production, and mtDNA levels are decreased in NPCs. These defects are linked to impaired neuronal proliferation and differentiation, contributing to reduced neuronal density [81].

In human iPSC-derived neurons from individuals with T21, mitochondrial dysfunction is marked by decreased MMP, structural abnormalities, and increased Ab accumulation. These changes correlate with increased DNA double-strand breakage, suggesting a connection between mitochondrial dysfunction and genomic instability [82]. Brain samples from DS fetuses (gestation week 22) and patients with DS-associated AD (DS-AD) exhibit reduced mtDNA copy numbers, increased somatic mtDNA mutations in the control region, and lower L-strand transcript levels, further linking mitochondrial dysfunction to neurodegeneration [83].

Proteomic analysis of DS fibroblasts revealed significant reductions in OXPHOS complex subunits and other mitochondrial proteins, with functional assays confirming defective mitochondrial respiration and ATP synthesis, reinforcing mitochondrial dysfunction as a hallmark of DS [84].

#### 3.4.2 HSA21 Gene-Mediated Dysregulation of Bioenergetics and Mitochondrial Biogenesis

Mitochondrial biogenesis, a highly coordinated process, integrates nuclear and mitochondrial genome expression to sustain cellular energy demands via multiple transcriptional networks. Nuclear respiratory factors (NRF-1 and NRF-2) regulate genes encoding OXPHOS complexes, mitochondrial import machinery, and heme biosynthesis while activating the mitochondrial transcription factors TFAM, TFB1M, and TFB2M required for mtDNA transcription [85]. Nuclear hormone receptors, including peroxisome proliferator-activated receptors (PPARs) and estrogen-related receptors (ERRs), regulate mitochondrial metabolism, dynamics, and lipid utilization. Central to this regulatory network is PGC-1α, a key transcriptional coactivator that integrates environmental and metabolic cues to enhance mitochondrial gene expression [86]. By interacting with NRFs, ERRs, and other transcription factors, PGC-1α coordinates mitochondrial adaptation to physiological stressors (e.g., caloric restriction, exercise, oxidative stress).

In DS, overexpression of HSA21 genes disrupts mitochondrial homeostasis by impairing bioenergetics and biogenesis. DYRK1A, an HSA21 gene product, phosphorylates nuclear factor of activated T cells (NFATc), preventing its nuclear translocation and suppressing *PPARGC1A*, which encodes PGC-1α, a key regulator of OXPHOS and mitochondrial biogenesis [87]. Overexpression of RCAN1, the human homolog of *Drosophila nebula*, contributes to mitochondrial dysfunction by reducing mitochondrial CIV activity, mtDNA content, and ATP levels. In ST14A rat striatal neurons and SH-SY5Y neuroblastoma cells, RCAN1 overexpression decreases mitochondrial mass and oxygen consumption, impairing NFATc activity [88, 89]. NRIP1, a transcriptional corepressor encoded on HSA21, negatively regulates oxidative metabolism and mitochondrial biogenesis. In DS fetal fibroblasts, NRIP1 overexpression suppresses PGC-1α, downregulating NEMG expression and exacerbating mitochondrial dysfunction [90]. SUMO3, another HSA21 gene product, modulates NRIP1 nuclear localization, enhancing its corepressor activity and inhibiting PGC-1α-dependent mitochondrial biogenesis [91]. SUMOylation of PGC-1α promotes nuclear export, attenuating its transcriptional activity and impairing mitochondrial adaptation [92]. *PKNOX1*, which encodes encoding homeodomain transcription factor PREP1, influences mitochondrial function. Muscle-specific PREP1 ablation enhances OXPHOS subunit expression, mitochondrial enzyme activity, and endurance capacity partly through upregulation of PGC-1α and reduced inhibition by p160 Mybbp1a, a PGC-1α inhibitor [93]. Finally, BACH1, a heme-binding transcription factor encoded on HSA21, suppresses electron transport chain (ETC) genes, reducing mitochondrial respiration. Its inhibition may improve mitochondrial function and metabolic reprogramming, possibly via PPARGC1A and NRF2, although the precise mechanisms remain unclear [94].

## 4. Mitochondrial Mechanisms Driving Accelerated Brain Aging in DS

### 4.1 AD-Like Pathology in DS

Individuals with DS have a markedly increased risk of developing AD. This strong association is evident in the presence of AD pathological hallmarks, including senile plaques, NFTs, and the progressive loss of cholinergic, noradrenergic, and serotonergic neurons. Nearly all individuals with DS exhibit AD-like neuropathology by age 40 [95], and the prevalence of dementia escalates with age, affecting approximately 8%, 55%, and 75% of individuals aged 35-49, 50-59, and 60 or older, respectively [96]. This trend suggests that DS and AD share common pathological mechanisms, which are probably driven by genetic factors linked to HSA21.

Despite these shared features, DS-AD and sporadic AD differ in onset, etiology, and clinical trajectory [97]. In DS, APP triplication due to trisomy 21 results in early and robust Aβ accumulation, often initiating in adolescence and preceding overt dementia by decades. Cognitive decline in DS unfolds on the background of lifelong intellectual disability, leading to an insidious and gradual deterioration. In contrast, sporadic AD typically presents in late adulthood with new-onset memory impairment in previously cognitively intact individuals, and Aβ deposition develops later in life under the influence of aging and genetic risk factors such as APOE ε4 [98]. Thus, DS-AD exemplifies a genetically determined, developmentally primed model of early-onset AD, whereas sporadic AD reflects a complex, age-dependent, and multifactorial disorder.

#### 4.1.1 Oxidative Stress and the “Two-Hit” Hypothesis

The “two-hit” hypothesis of AD posits that while oxidative stress and mitotic signaling abnormalities are independent initiators of neurodegeneration, their convergence is necessary and sufficient to drive AD pathogenesis [99, 100]. This hypothesis suggests that particularly in people with a genetic predisposition, neurons under chronic stress divert their cellular resources toward compensatory mechanisms, leaving them vulnerable to subsequent pathological insults. An initial oxidative burden weakens neuronal defenses, heightening susceptibility to further neurodegenerative processes.

This hypothesis is particularly relevant to DS, where oxidative stress emerges early due to the gene dosage effects of T21. The early and persistent overexpression of *SOD1* and *APP* in DS establishes a pro-oxidative cellular environment, lowering neuronal resilience to additional neurodegenerative insults [65, 96]. The accumulation of oxidative damage in DS neurons parallels that in AD, reinforcing the role of oxidative stress as a central driver of neurodegeneration in both conditions.

While oxidative stress is generally viewed as a driver of neuronal dysfunction in DS, some studies suggest that mitochondrial suppression may also represent an adaptive response to chronic redox imbalance. In vitro experiments have shown that DS neurons downregulate mitochondrial respiration and ATP production to limit ROS generation, and that forced enhancement of mitochondrial activity can paradoxically exacerbate oxidative damage and trigger apoptosis [12]. These findings suggest that not all mitochondrial deficits in DS are purely maladaptive but may instead reflect a compensatory shift in cellular metabolism aimed at preserving redox homeostasis under constitutive stress conditions.

#### 4.1.2 A Converging Pathway: The Role of APP Gene Dose

Triplication of HSA21, which includes *APP*, is among the most compelling links between DS and AD. The gene dosage effect results in a 1.5-fold increase in APP expression, leading to excessive production of Aβ, a key pathological hallmark of AD [101]. In DS, amyloid pathology develops significantly earlier than in sporadic AD. Intracellular Aβ accumulation begins within enlarged endosomes, preceding the formation of extracellular plaques. This amyloid deposition follows a spatiotemporal pattern similar to that in AD but occurs decades earlier, with Aβ aggregation beginning in adolescence and NFT formation becoming apparent by the fourth decade of life [97].

Aβ oligomers are particularly neurotoxic, contributing to cognitive decline by impairing synaptic transmission, disrupting neurotransmitter signaling, and inducing synaptic loss [102, 103]. Overproduction of the aggregation-prone Aβ42 variant accelerates amyloid deposition, and DS brains exhibit denser Aβ plaques than those seen in sporadic AD [104]. However, Aβ toxicity is not isolated but catalyzes a cascade of pathological processes that drive neurodegeneration.

Mitochondrial dysfunction is the key downstream effects of *APP* overexpression and Aβ accumulation. Aβ disrupts electron transport chain activity, increases ROS production, and impairs ATP generation, leading to severe neuronal energy deficits and creating a self-perpetuating cycle of oxidative stress and neuronal damage, further exacerbating AD-like pathology. Notably, targeting *APP* expression with antisense oligonucleotides (ASOs) has been shown to restore mitochondrial function in DS astrocytes, highlighting *APP* dosage as a key upstream driver of mitochondrial and neurodegenerative pathology—and a promising therapeutic target for both DS and AD [105].

#### 4.1.3 Additional Chromosome 21 Genes Contributing to AD Pathology in DS

Other HSA21 genes also contribute to neurodegeneration. Chronic *RCAN1* overexpression inhibits calcineurin, leading to tau hyperphosphorylation and early NFT formation [106]. Additionally, *RCAN1* has been implicated in upregulating expression of GSK-3β, a key kinase involved in tau pathology to exacerbate NFT formation, though additional studies of this relationship are needed. Aβ can directly induce *RCAN1* expression, reinforcing a vicious cycle of Aβ toxicity, tau dysregulation, and neuronal damage [107]. Another key contributor is S100B, a calcium-binding protein aberrantly overexpressed in DS astrocytes. Elevated S100B levels correlate with age and are associated with increased Aβ plaque formation, suggesting a role in AD-like pathology [108]. *DYRK1A* has been implicated in tau hyperphosphorylation and abnormal cell cycle re-entry, further linking DS with AD pathogenesis [109].

### 4.2 Mitochondrial Dysfunction as a Driver of Early Cellular Senescence in DS

Cellular senescence, first described by Hayflick in the 1960s, refers to the limited proliferative potential of human fibroblasts cultured in vitro, ultimately leading to irreversible cell cycle arrest [110]. It is now recognized as a homeostatic biological process triggered by environmental stress, with crucial roles in development, tissue remodeling, and tumor suppression. However, dysregulated senescence contributes to tissue regenerative decline, chronic disease, and aging [111]. The accumulation of senescent cells is a hallmark of aging implicated in the onset and progression of age-associated diseases [112].

Mitochondria are central regulators of cellular senescence, sustaining ROS production, amplifying DNA damage, and modulating pro-inflammatory signaling. The ATM-Akt-mTORC1-PGC-1β signaling axis plays a key role in mitochondrial biogenesis, which reinforces SASP, a major driver of age-related pathology, under chronic oxidative stress. Reducing mitochondrial content via mTORC1 inhibition or PGC-1β deletion mitigates senescence in aging tissues, highlighting mitochondria as a crucial regulatory hub in senescence progression. Recent evidence suggests that, like mitotic cells, post-mitotic neurons can acquire a senescence-like state, further implicating senescence in DS-associated neurodegeneration [113, 114].

#### 4.2.1 Cellular Senescence in DS

Features of accelerated cellular senescence are evident in DS pathology and parallel those in early-onset AD. Leukocytes from individuals with DS exhibit significantly elevated senescence-associated β-galactosidase (SA-β-gal) activity, indicating widespread lysosomal dysfunction and premature cellular aging [115]. Hippocampal tissue from Ts65Dn DS mouse models shows an increased presence of SA-β-gal-positive cells and elevated levels of pro-inflammatory cytokines such as interleukin-17A, interleukin-1β, and interferon-γ, further linking chronic inflammation and senescence [116]. DS fibroblasts are deficient in DNA polymerase β, a key enzyme involved in BER, which compromises genomic integrity and exacerbates premature senescence [117]. NPCs derived from DS-iPSCs display substantial nuclear architectural alterations, including chromosomal introversion, disrupted lamina-associated domains (LADs), and global chromatin accessibility changes. These alterations correlate with the transcriptional dysregulation characteristic of senescent cells, evidenced by upregulated senescence markers (p16, p21), downregulated proliferation-associated genes, and enriched pro-inflammatory pathways [118]. DS mouse models also exhibit accelerated senescence across multiple stem cell lineages, further contributing to age-related functional decline. In Ts65Dn mice, overexpression of Usp16, a deubiquitinase regulating chromatin remodeling and cell cycle progression, leads to impaired hematopoietic stem cell self-renewal, a threefold reduction in stem cell frequency, and defective multilineage engraftment [119]. Skeletal muscle stem cells in DS exhibit increased DNA damage and diminished regenerative capacity, further demonstrating the widespread impact of senescence on tissue function [120].

#### 4.2.2 Interplay Between Oxidative Stress, Mitochondrial Dysfunction, and Early Cellular Senescence in DS

Evidence suggests that senescence-associated defects arise early in DS development, significantly impacting neurogenesis and overall cellular homeostasis. In DS fetuses, NPCs exhibit impaired neurogenesis due to delayed cell cycle progression and reduced proliferation. Accumulation of cells in the G2 phase, which indicates a premature senescence-like state, has been observed in both DS fetal brains and Ts65Dn mice [121]. Telomere attrition further exacerbates this phenomenon: DS fetal amniocytes display significantly shorter telomeres and upregulation of *TERC*, which encodes the RNA component of telomerase. This dysregulation suggests an inherent predisposition to genomic instability, which may underlie both hematopoietic malignancies and neurodegeneration in DS [122]. DS placental trophoblasts exhibit an increased prevalence of senescence-associated heterochromatin foci, an epigenetic marker of cellular senescence, as early as the second trimester, suggesting that trophoblast senescence could be a potential early biomarker of T21 during pregnancy [123].

Premature senescence is centered around oxidative stress, which is particularly pronounced in DS due to impaired mitochondrial function. DS-derived fibroblasts exhibit excessive ROS accumulation, increased protein oxidation, and ATP depletion, which collectively activate key senescence markers, including lysosomal SA-β-gal and p21. These cellular stressors contribute to accelerated aging, reinforcing the premature senescence observed in DS [124]. In DS mouse models, hippocampal oxidative stress correlates with heightened Aβ expression, tau hyperphosphorylation, and widespread cellular senescence across key brain regions such as the dentate gyrus, CA1, CA3, and hilus [116].

**Figure 2.**
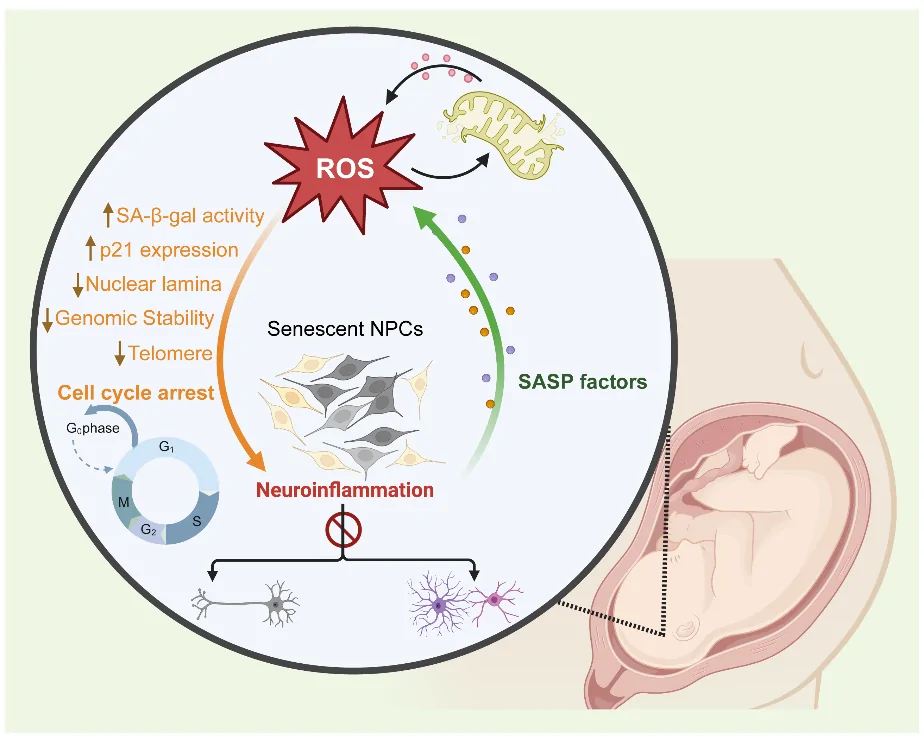
**Early Onset of Cellular Senescence and Neuroinflammation in DS**. Mitochondrial dysfunction and ROS drive the premature onset of cellular senescence as early as the prenatal stage, particularly in neural progenitor cells (NPCs). Hallmarks of senescence include heightened lysosomal SA-β-gal activity, genomic instability, elevated p21 expression, severe deficits in antioxidant defenses, mitochondrial impairment, nuclear lamina disruption, telomere shortening, and cell cycle arrest. The accumulation of senescent cells triggers a proinflammatory environment through the senescence-associated secretory phenotype (SASP), leading to chronic neuroinflammation and impaired neurogenesis. This persistent inflammatory state contributes to the progression of brain aging and neurodegeneration in DS.

Beginning in the prenatal stage, oxidative stress, mitochondrial dysfunction, and impaired antioxidant defenses synergistically drive premature cellular senescence, establishing a trajectory that accelerates neurodegenerative pathology. Accumulated senescent cells foster a proinflammatory environment through the SASP, leading to chronic neuroinflammation and impairing neurogenesis (Fig. 2). As individuals with DS age, persistent oxidative stress and mitochondrial deficits exacerbate senescence, coinciding with the emergence of AD pathology and progressive cognitive decline. This intricate interplay between oxidative stress, mitochondrial dysfunction, and cellular senescence highlights a critical pathogenic axis in DS, underscoring the urgent need for targeted therapeutic strategies aimed at restoring mitochondrial homeostasis and mitigating oxidative damage.

## 5. Therapeutic Strategies Targeting Mitochondrial Dysfunction in DS

Mitochondrial dysfunction is a central driver of accelerated aging and neurodegeneration in DS. Dysregulated mitochondrial energy metabolism lead to increased oxidative stress, chronic inflammation, and neuronal damage, contributing to systemic aging and cognitive decline. Therapeutic interventions targeting mitochondrial function may be potential strategies to mitigate neurodegenerative processes and improve cognitive outcomes in DS. In the following sections, we summarize major mechanistic targets and highlight representative agents with preclinical or early translational relevance.

### 5.1 Antioxidant Therapies

Oxidative stress is an early, persistent feature of DS that negatively impacts brain development and worsens with age, ultimately contributing to AD-like neuropathology and accelerated aging. Antioxidant therapies have been explored as potential interventions to counteract oxidative stress, restore mitochondrial function, and improve cognition in individuals with DS.

Vitamin E, a potent lipid-soluble antioxidant, protects cellular membranes from lipid peroxidation. Studies in DS mouse models have shown that long-term vitamin E supplementation reduces oxidative stress, improves cognitive performance, and prevents cholinergic neuronal degeneration, suggesting a potential role in mitigating age-related neuropathology [125]. However, clinical trials in older adults with DS have not demonstrated significant cognitive benefits, possibly due to limited Central Nervous System (CNS) penetration and the insufficient potency of α-tocopherol alone, a commonly used isoform of vitamin E [126, 127].

Vitamin C, a water-soluble antioxidant, complements vitamin E by regenerating its reduced form, enhancing its overall antioxidant efficacy. Clinical trials involving children and adolescents with DS have demonstrated that combined vitamin E and C supplementation reduces systemic oxidative stress, restores glutathione levels, and improves biomarkers of oxidative damage such as thiobarbituric acid reactive substances [128, 129].

Coenzyme Q10 (CoQ10), a key mitochondrial electron transport chain component, is a potent antioxidant that facilitates electron transfer. In children and adolescents with DS, long-term CoQ10 supplementation (4 mg/kg/day for 20 months) significantly reduced oxidative DNA damage in peripheral leukocytes, particularly in younger individuals [130]. However, extended supplementation (4 years) at the same dose had no significant impact on overall DNA and RNA oxidation, suggesting that further optimization of CoQ10 formulations and dosages is needed [131].

Children with DS have a high prevalence (60%-95%) of obstructive sleep apnea (OSA), which exacerbates cognitive impairment and behavioral deficits [132]. These children have lower plasma concentrations of melatonin, a hormone traditionally associated with sleep regulation, increasing their vulnerability to oxidative damage [133]. In Ts65Dn DS mice, chronic melatonin administration reduced hippocampal lipid peroxidation and protein carbonylation while normalizing SA-β-gal-positive cell density, indicating reduced cellular senescence [134, 135]. Melatonin was found to improve spatial learning and memory, suggesting its neuroprotective potential [135]. Given its established use in clinical settings for sleep disturbances and its favorable safety record in humans, melatonin holds promise as a candidate for future DS-targeted clinical trials.

EGCG, a polyphenol from green tea, exhibits stronger antioxidant properties than vitamins E and C due to its ability to stabilize phenoxy radicals through multiple resonance structures. Unlike other antioxidants, EGCG selectively accumulates in mitochondria, scavenging free radicals at the source of oxidative damage [136, 137]. In DS cellular models, EGCG enhances mitochondrial oxidative phosphorylation by activating the cAMP/PKA and Sirtuin 1 (Sirt1)/PGC-1α signaling pathways, rescuing CI and ATP synthase activities while reducing excessive ROS production [138]. In a phase II randomized controlled trial (TESDAD), 12-month EGCG supplementation improved visual recognition memory and adaptive behavior in young adults with DS, suggesting translational potential for early intervention [139].

Notably, some studies have reported inconsistent outcomes across antioxidant trials and biomarker analyses in DS, with variability linked to age, cellular context, and the specific oxidative pathways assessed [65]. These inconsistencies highlight the need for larger, well-controlled trials to confirm efficacy, define optimal formulations, and assess long-term safety across age groups.

### 5.2 Inducers of Mitochondrial Biogenesis

Mitochondrial function requires efficient mitochondrial biogenesis, a process regulated by key transcriptional and metabolic pathways. Enhancing mitochondrial biogenesis is a promising therapeutic strategy for restoring mitochondrial homeostasis and improving neuronal function in DS. Mitochondrial biogenesis is controlled by the AMP-activated protein kinase (AMPK)-Sirt1-PGC-1α axis. Activation of AMPK, a key energy sensor, leads to downstream phosphorylation events, promoting ATP synthesis while inhibiting ATP-consuming processes. Activated AMPK enhances Sirt1 activity, which in turn stimulates PGC-1α, a master regulator of mitochondrial biogenesis.

EGCG can enhance mitochondrial respiratory chain function and promote mitochondrial biogenesis in DS models. In Ts65Dn mice, EGCG treatment (20 μM) improved NPC proliferation by restoring mitochondrial bioenergetics [140]. EGCG enhances CI activity in fibroblasts from individuals with DS by modulating cAMP levels, activating PKA, and restoring NDUFS4 subunit function, ultimately improving mitochondrial efficiency [138].

Resveratrol, a stilbenoid-class polyphenol, similarly rescues mitochondrial dysfunction in DS. In DS models, resveratrol (10 μM) restores OXPHOS and mitochondrial biogenesis by activating the AMPK-Sirt1-PGC-1α axis to improve neurogenesis and cognitive function [140]. Resveratrol supplementation for 4 weeks has been shown to decrease oxidative stress and ameliorate age-related metabolic dysfunction in patients with type 2 diabetes mellitus (T2DM) [141]. However, these drugs have not yet been validated in DS-specific clinical trials, and their safety profiles, especially in pediatric populations, warrant careful consideration.

Metformin, a widely used medication for diabetes, has emerged as a candidate anti-aging intervention due to its ability to modulate key longevity pathways. Notably, large-scale clinical trials such as the Targeting Aging with Metformin (TAME) study aim to evaluate its efficacy in delaying multiple age-related diseases and promoting healthy aging [142]. Mechanistically, metformin enhances PGC-1α expression and promotes mitochondrial biogenesis. Studies in DS fibroblasts suggest that metformin restores mitochondrial homeostasis, positioning it as a promising candidate for repurposing in DS therapy [70].

### 5.3 Modulators of Mitochondrial Dynamics

Mitochondrial homeostasis depends on the balance between fusion and fission, which are disrupted in DS. Excessive mitochondrial fission and reduced fusion lead to mitochondrial fragmentation, impaired energy production, and increased oxidative stress [143]. Enhancing mitochondrial fusion by upregulating mitofusins (MFN1 and MFN2) can restore mitochondrial integrity, improve respiratory efficiency, and reduce apoptosis. Inhibiting excessive fission by targeting dynamin-related protein 1 (DRP1) can help preserve mitochondrial function and neuronal survival [144].

Metformin was shown to rescue mitochondrial dynamics in DS fetal fibroblasts by upregulating OPA1 and MFN2, restoring mitochondrial fusion, and reducing fragmentation [70]. Similarly, Mdivi-1, a selective DRP1 inhibitor, rescued mitochondrial network integrity in hippocampal NPCs from Ts65Dn mice, mitigating excessive fission and improving mitochondrial bioenergetics [71]. While Mdivi-1 is currently limited to preclinical research, DRP1 remains a pharmacologically tractable target under investigation in broader neurodegenerative contexts such as AD and PD [142].

Pioglitazone (PGZ), a PPARγ agonist, restores mitochondrial organization in DS fibroblasts by modulating mitochondrial dynamics. PGZ increases OPA1 and MFN1 expression while downregulating DRP1, increasing mitochondrial network interconnectivity [145]. EGCG also restored mitochondrial dynamics by reducing calcium overload, balancing fission and fusion, and improving mitochondrial morphology and function in hippocampal neurons from DS models [146].

### 5.4 Enhancers of Mitophagy

Enhancing mitophagy is a promising therapeutic strategy for alleviating mitochondrial dysfunction in DS. Rapamycin, a potent mTOR inhibitor, is a well-established autophagy inducer that promotes cellular homeostasis by suppressing mTORC1 activity, enhancing autophagosome formation and mitigating age-related and disease-associated cellular dysfunction [147]. In Ts65Dn mice, 12-week rapamycin treatment restored mTOR signaling, increased LC3-II levels, and reduced oxidative stress markers, suggesting improved autophagy and mitophagy regulation [148]. However, systemic administration of rapamycin is associated with side effects such as immunosuppression and metabolic disturbance, limiting its long-term use in vulnerable populations like DS.

Beyond classical autophagy inducers, partial inhibition of DRP1 has also been shown to enhance mitophagy and improve mitochondrial quality in AD models [149]. Additional compounds such as Urolithin A and Tomatidine have demonstrated mitophagy-promoting effects via the PINK1-Parkin and AMPK-mTOR pathways, respectively, in aging and AD models [150]. Urolithin A has completed early-phase human trials and was shown to induce mitophagy-related gene expression in skeletal muscle [151], suggesting translational potential for conditions involving CNS mitochondrial dysfunction.

These observations are consistent with broader findings showing that defective mitophagy driven by oxidative stress, impaired DNA repair, and telomere shortening contributes to neurodegeneration in aging and AD [152]. While these agents have not yet been tested in DS, their mechanisms may be relevant given the shared mitochondrial defects, warranting further investigation in DS-specific systems.

### 5.5 Summary and Clinical Outlook

Together, these therapeutic strategies reflect the multifaceted nature of mitochondrial dysfunction in DS and highlight diverse molecular routes for intervention, including antioxidant supplementation, mitochondrial biogenesis inducers, dynamics modulators, and mitophagy enhancers. Most candidates remain at the preclinical stage, and translational challenges persist, including the scarcity of DS-focused clinical studies, age-related variability in treatment response, and the need for CNS-penetrant compounds and robust biomarkers. Addressing these barriers will be essential for advancing mitochondrial-targeted therapies toward clinical application in DS.

Senolytic therapies, which selectively eliminate senescent cells, have emerged as a novel strategy to target mitochondrial-driven aging processes [153]. Agents such as dasatinib plus quercetin (D+Q) and Navitoclax (ABT-263) act through mitochondrial apoptotic pathways or inhibit anti-apoptotic BCL-2 proteins [151]. In DS models, D+Q has been shown to alleviate transcriptional and cellular dysfunction in neural progenitor cells, suggesting potential therapeutic relevance [118]. However, Navitoclax’s hematologic toxicity, particularly thrombocytopenia [154], raises safety concerns for clinical application, especially in pediatric or vulnerable DS populations. To overcome such limitations, mitochondria-targeted senolytics like MitoTam have recently been developed, demonstrating high selectivity for senescent cells and no major toxicity observed in preclinical models [155].

In addition to age, sex-based biological differences may influence mitochondrial function, neuroinflammation, and treatment response. Recent studies in DS-AD suggest that although overall AD penetrance and biomarker trajectories are largely similar between sexes, females carrying the APOE ε4 allele tend to exhibit earlier symptom onset, poorer episodic memory, and reduced hippocampal volume compared to males, indicating a potential interaction between sex and APOE genotype [156]. Transcriptomic profiling also reveals consistent upregulation of inflammatory and glial genes in DS-AD females, particularly in late-stage disease, suggesting heightened neuroinflammatory vulnerability [157]. These findings underscore the need to incorporate sex as a biological variable in future DS-specific clinical trials to improve therapeutic stratification and precision.

## 6. Conclusion

Mitochondrial imbalance is a defining feature of DS that fundamentally disrupts OXPHOS, mitochondrial dynamics, and quality control systems. These imbalances arise from HSA21 gene dosage effects and widespread transcriptional dysregulation, leading to persistent oxidative stress, energy deficits, and accumulation of dysfunctional mitochondria. Such impairments not only increase neuronal vulnerability but also exacerbate cellular senescence and neuroinflammation, reinforcing a pathological cycle that accelerates AD-like neurodegeneration in DS.

Beyond DS, the concept of mitochondrial imbalance represents a broader mechanism underlying neurodegenerative disorders. In conditions such as AD, amyotrophic lateral sclerosis, Parkinson’s disease, and Huntington’s disease, similar disruption of mitochondrial homeostasis underscores its role in neurodegenerative diseases [158-161]. Given its fundamental role in neuronal function and aging, therapeutic interventions targeting mitochondrial imbalance through strategies that enhance biogenesis, stabilize dynamics, and promote mitophagy are promising.

As a model for early-onset neurodegeneration, DS provides a unique window into mitochondrial pathology, offering insights applicable to other neurological disorders [97]. Addressing both systemic- and cellular-level mitochondrial imbalance will be essential for developing precision therapies that restore cellular resilience. Interdisciplinary collaboration will be crucial for translating these mechanistic insights into effective treatments for DS and a wider array of neurodegenerative diseases.

## References

[1] Zong Y, Li H, Liao P, Chen L, Pan Y, Zheng Y, et al. (2024). Mitochondrial dysfunction: mechanisms and advances in therapy. Signal Transduct Target Ther, 9:124. 10.1038/s41392-024-01839-8 PMC11094169 38744846

[2] Bustamante-Barrientos FA, Luque-Campos N, Araya MJ, Lara-Barba E, de Solminihac J, Pradenas C, et al. (2023). Mitochondrial dysfunction in neurodegenerative disorders: Potential therapeutic application of mitochondrial transfer to central nervous system-residing cells. J Transl Med, 21:613. 10.1186/s12967-023-04493-w PMC10493034 37689642

[3] Perrone L (2023). Editorial: Mitochondrial dysfunction as a target in neurodegenerative diseases. Front Mol Neurosci, 16:1271175. 10.3389/fnmol.2023.1271175 PMC10523779 37771558

[4] Rueda Revilla N and Martinez-Cue C (2020). Antioxidants in Down Syndrome: From Preclinical Studies to Clinical Trials. Antioxidants (Basel), 9. 10.3390/antiox9080692 PMC7464577 32756318

[5] Baburamani AA, Patkee PA, Arichi T and Rutherford MA (2019). New approaches to studying early brain development in Down syndrome. Dev Med Child Neurol, 61:867-879. 10.1111/dmcn.14260 PMC6618001 31102269

[6] Bayona-Bafaluy MP, Garrido-Perez N, Meade P, Iglesias E, Jimenez-Salvador I, Montoya J, et al. (2021). Down syndrome is an oxidative phosphorylation disorder. Redox Biol, 41:101871. 10.1016/j.redox.2021.101871 PMC7859316 33540295

[7] Head E, Powell D, Gold BT and Schmitt FA (2012). Alzheimer's Disease in Down Syndrome. Eur J Neurodegener Dis, 1:353-364. PMC4184282 25285303

[8] Wiseman FK, Al-Janabi T, Hardy J, Karmiloff-Smith A, Nizetic D, Tybulewicz VL, et al. (2015). A genetic cause of Alzheimer disease: mechanistic insights from Down syndrome. Nat Rev Neurosci, 16:564-74. 10.1038/nrn3983 PMC4678594 26243569

[9] Tan KL, Lee HC, Cheah PS and Ling KH (2023). Mitochondrial Dysfunction in Down Syndrome: From Pathology to Therapy. Neuroscience, 511:1-12. 10.1016/j.neuroscience.2022.12.003 36496187

[10] Perluigi M, Di Domenico F and Buttterfield DA (2014). Unraveling the complexity of neurodegeneration in brains of subjects with Down syndrome: insights from proteomics. Proteomics Clin Appl, 8:73-85. 10.1002/prca.201300066 PMC3965623 24259517

[11] Ganguly BB and Kadam NN (2023). Therapeutics for mitochondrial dysfunction-linked diseases in Down syndrome. Mitochondrion, 68:25-43. 10.1016/j.mito.2022.11.003 36371073

[12] Helguera P, Seiglie J, Rodriguez J, Hanna M, Helguera G and Busciglio J (2013). Adaptive downregulation of mitochondrial function in down syndrome. Cell Metab, 17:132-40. 10.1016/j.cmet.2012.12.005 PMC3580189 23312288

[13] Gensous N, Bacalini MG, Franceschi C and Garagnani P (2020). Down syndrome, accelerated aging and immunosenescence. Semin Immunopathol, 42:635-645. 10.1007/s00281-020-00804-1 PMC7666319 32705346

[14] Lopez-Otin C, Blasco MA, Partridge L, Serrano M and Kroemer G (2023). Hallmarks of aging: An expanding universe. Cell, 186:243-278. 10.1016/j.cell.2022.11.001 36599349

[15] Tayebati SK, Cecchi A, Martinelli I, Carboni E and Amenta F (2019). Pharmacotherapy of Down's Syndrome: When and Which? CNS Neurol Disord Drug Targets, 18:750-757. 10.2174/1871527318666191114092924 31724517

[16] Peng L, Baradar AA, Aguado J and Wolvetang E (2023). Cellular senescence and premature aging in Down Syndrome. Mech Ageing Dev, 212:111824. 10.1016/j.mad.2023.111824 37236373

[17] Kozlov G, Franceschi C and Vedunova M (2024). Intricacies of aging and Down syndrome. Neurosci Biobehav Rev, 164:105794. 10.1016/j.neubiorev.2024.105794 38971514

[18] Horvath S, Garagnani P, Bacalini MG, Pirazzini C, Salvioli S, Gentilini D, et al. (2015). Accelerated epigenetic aging in Down syndrome. Aging Cell, 14:491-5. 10.1111/acel.12325 PMC4406678 25678027

[19] Aivazidis S, Coughlan CM, Rauniyar AK, Jiang H, Liggett LA, Maclean KN, et al. (2017). The burden of trisomy 21 disrupts the proteostasis network in Down syndrome. PLoS One, 12:e0176307. 10.1371/journal.pone.0176307 PMC5400264 28430800

[20] Bordi M, Darji S, Sato Y, Mellen M, Berg MJ, Kumar A, et al. (2019). mTOR hyperactivation in Down Syndrome underlies deficits in autophagy induction, autophagosome formation, and mitophagy. Cell Death Dis, 10:563. 10.1038/s41419-019-1752-5 PMC6646359 31332166

[21] Isaev NK, Genrikhs EE, Oborina MV and Stelmashook EV (2018). Accelerated aging and aging process in the brain. Rev Neurosci, 29:233-240. 10.1515/revneuro-2017-0051 29150992

[22] Down JL (1995). Observations on an ethnic classification of idiots. 1866. Ment Retard, 33:54-6. 7707939

[23] Lejeune J, Gautier M and Turpin R (1959). [Study of somatic chromosomes from 9 mongoloid children]. C R Hebd Seances Acad Sci, 248:1721-2. 13639368

[24] Gardiner K and Davisson M (2000). The sequence of human chromosome 21 and implications for research into Down syndrome. Genome Biol, 1:REVIEWS0002. 10.1186/gb-2000-1-2-reviews0002 PMC138845 11178230

[25] Epstein CJ, Avraham KB, Lovett M, Smith S, Elroy-Stein O, Rotman G, et al. (1987). Transgenic mice with increased Cu/Zn-superoxide dismutase activity: animal model of dosage effects in Down syndrome. Proc Natl Acad Sci U S A, 84:8044-8. 10.1073/pnas.84.22.8044 PMC299473 2960971

[26] Davisson MT, Schmidt C and Akeson EC (1990). Segmental trisomy of murine chromosome 16: a new model system for studying Down syndrome. Prog Clin Biol Res, 360:263-80. 2147289

[27] Lyle R, Gehrig C, Neergaard-Henrichsen C, Deutsch S and Antonarakis SE (2004). Gene expression from the aneuploid chromosome in a trisomy mouse model of down syndrome. Genome Res, 14:1268-74. 10.1101/gr.2090904 PMC442141 15231743

[28] Kahlem P, Sultan M, Herwig R, Steinfath M, Balzereit D, Eppens B, et al. (2004). Transcript level alterations reflect gene dosage effects across multiple tissues in a mouse model of down syndrome. Genome Res, 14:1258-67. 10.1101/gr.1951304 PMC442140 15231742

[29] Vilardell M, Rasche A, Thormann A, Maschke-Dutz E, Perez-Jurado LA, Lehrach H, et al. (2011). Meta-analysis of heterogeneous Down Syndrome data reveals consistent genome-wide dosage effects related to neurological processes. BMC Genomics, 12:229. 10.1186/1471-2164-12-229 PMC3110572 21569303

[30] Antonarakis SE (2017). Down syndrome and the complexity of genome dosage imbalance. Nat Rev Genet, 18:147-163. 10.1038/nrg.2016.154 28029161

[31] Fullerton HJ, Ditelberg JS, Chen SF, Sarco DP, Chan PH, Epstein CJ, et al. (1998). Copper/zinc superoxide dismutase transgenic brain accumulates hydrogen peroxide after perinatal hypoxia ischemia. Ann Neurol, 44:357-64. 10.1002/ana.410440311 9749602

[32] Manczak M, Mao P, Calkins MJ, Cornea A, Reddy AP, Murphy MP, et al. (2010). Mitochondria-targeted antioxidants protect against amyloid-beta toxicity in Alzheimer's disease neurons. J Alzheimers Dis, 20 Suppl 2:S609-31. 10.3233/JAD-2010-100564 PMC3072711 20463406

[33] Bartley MG, Marquardt K, Kirchhof D, Wilkins HM, Patterson D and Linseman DA (2012). Overexpression of amyloid-beta protein precursor induces mitochondrial oxidative stress and activates the intrinsic apoptotic cascade. J Alzheimers Dis, 28:855-68. 10.3233/JAD-2011-111172 PMC4679200 22133762

[34] Chang KT and Min KT (2005). Drosophila melanogaster homolog of Down syndrome critical region 1 is critical for mitochondrial function. Nat Neurosci, 8:1577-85. 10.1038/nn1564 16222229

[35] Sun X, Wu Y, Herculano B and Song W (2014). RCAN1 overexpression exacerbates calcium overloading-induced neuronal apoptosis. PLoS One, 9:e95471. 10.1371/journal.pone.0095471 PMC3994074 24751678

[36] Walter C, Marada A, Suhm T, Ernsberger R, Muders V, Kucukkose C, et al. (2021). Global kinome profiling reveals DYRK1A as critical activator of the human mitochondrial import machinery. Nat Commun, 12:4284. 10.1038/s41467-021-24426-9 PMC8277783 34257281

[37] Helguera P, Pelsman A, Pigino G, Wolvetang E, Head E and Busciglio J (2005). ets-2 promotes the activation of a mitochondrial death pathway in Down's syndrome neurons. J Neurosci, 25:2295-303. 10.1523/JNEUROSCI.5107-04.2005 PMC6726094 15745955

[38] Qiu JJ, Liu YN, Ren ZR and Yan JB (2017). Dysfunctions of mitochondria in close association with strong perturbation of long noncoding RNAs expression in down syndrome. Int J Biochem Cell Biol, 92:115-120. 10.1016/j.biocel.2017.09.017 28965985

[39] Quinones-Lombrana A and Blanco JG (2015). Chromosome 21-derived hsa-miR-155-5p regulates mitochondrial biogenesis by targeting Mitochondrial Transcription Factor A (TFAM). Biochim Biophys Acta, 1852:1420-7. 10.1016/j.bbadis.2015.04.004 PMC4433801 25869329

[40] Izzo A, Manco R, de Cristofaro T, Bonfiglio F, Cicatiello R, Mollo N, et al. (2017). Overexpression of Chromosome 21 miRNAs May Affect Mitochondrial Function in the Hearts of Down Syndrome Fetuses. Int J Genomics, 2017:8737649. 10.1155/2017/8737649 PMC5605795 29057256

[41] Markesbery WR and Lovell MA (2007). Damage to lipids, proteins, DNA, and RNA in mild cognitive impairment. Arch Neurol, 64:954-6. 10.1001/archneur.64.7.954 17620484

[42] Perluigi M, di Domenico F, Fiorini A, Cocciolo A, Giorgi A, Foppoli C, et al. (2011). Oxidative stress occurs early in Down syndrome pregnancy: A redox proteomics analysis of amniotic fluid. Proteomics Clin Appl, 5:167-78. 10.1002/prca.201000121 21360684

[43] Segal BH, Grimm MJ, Khan AN, Han W and Blackwell TS (2012). Regulation of innate immunity by NADPH oxidase. Free Radic Biol Med, 53:72-80. 10.1016/j.freeradbiomed.2012.04.022 PMC3377837 22583699

[44] Sandalio LM, Rodriguez-Serrano M, Romero-Puertas MC and del Rio LA (2013). Role of peroxisomes as a source of reactive oxygen species (ROS) signaling molecules. Subcell Biochem, 69:231-55. 10.1007/978-94-007-6889-5_13 23821152

[45] Balaban RS, Nemoto S and Finkel T (2005). Mitochondria, oxidants, and aging. Cell, 120:483-95. 10.1016/j.cell.2005.02.001 15734681

[46] Busciglio J, Pelsman A, Wong C, Pigino G, Yuan M, Mori H, et al. (2002). Altered metabolism of the amyloid beta precursor protein is associated with mitochondrial dysfunction in Down's syndrome. Neuron, 33:677-88. 10.1016/s0896-6273(02)00604-9 11879646

[47] Di Domenico F, Pupo G, Mancuso C, Barone E, Paolini F, Arena A, et al. (2015). Bach1 overexpression in Down syndrome correlates with the alteration of the HO-1/BVR-a system: insights for transition to Alzheimer's disease. J Alzheimers Dis, 44:1107-20. 10.3233/JAD-141254 PMC4677575 25391381

[48] Esposito G, Imitola J, Lu J, De Filippis D, Scuderi C, Ganesh VS, et al. (2008). Genomic and functional profiling of human Down syndrome neural progenitors implicates S100B and aquaporin 4 in cell injury. Hum Mol Genet, 17:440-57. 10.1093/hmg/ddm322 17984171

[49] Brooksbank BW and Balazs R (1984). Superoxide dismutase, glutathione peroxidase and lipoperoxidation in Down's syndrome fetal brain. Brain Res, 318:37-44. 10.1016/0165-3806(84)90060-9 6237715

[50] Sinha S (2005). Anti-oxidant gene expression imbalance, aging and Down syndrome. Life Sci, 76:1407-26. 10.1016/j.lfs.2004.10.026 15670619

[51] Di Carlo M, Giacomazza D, Picone P, Nuzzo D and San Biagio PL (2012). Are oxidative stress and mitochondrial dysfunction the key players in the neurodegenerative diseases? Free Radic Res, 46:1327-38. 10.3109/10715762.2012.714466 22817279

[52] Gimeno A, Garcia-Gimenez JL, Audi L, Toran N, Andaluz P, Dasi F, et al. (2014). Decreased cell proliferation and higher oxidative stress in fibroblasts from Down Syndrome fetuses. Preliminary study. Biochim Biophys Acta, 1842:116-25. 10.1016/j.bbadis.2013.10.014 24184606

[53] Garlet TR, Parisotto EB, de Medeiros Gda S, Pereira LC, Moreira EA, Dalmarco EM, et al. (2013). Systemic oxidative stress in children and teenagers with Down syndrome. Life Sci, 93:558-63. 10.1016/j.lfs.2013.08.017 24004546

[54] Sanchez-Font MF, Sebastia J, Sanfeliu C, Cristofol R, Marfany G and Gonzalez-Duarte R (2003). Peroxiredoxin 2 (PRDX2), an antioxidant enzyme, is under-expressed in Down syndrome fetal brains. Cell Mol Life Sci, 60:1513-23. 10.1007/s00018-003-3048-1 PMC11138650 12943237

[55] Meguid NA, Dardir AA, El-Sayed EM, Ahmed HH, Hashish AF and Ezzat A (2010). Homocysteine and oxidative stress in Egyptian children with Down syndrome. Clin Biochem, 43:963-7. 10.1016/j.clinbiochem.2010.04.058 20450901

[56] Saghazadeh A, Mahmoudi M, Dehghani Ashkezari A, Oliaie Rezaie N and Rezaei N (2017). Systematic review and meta-analysis shows a specific micronutrient profile in people with Down Syndrome: Lower blood calcium, selenium and zinc, higher red blood cell copper and zinc, and higher salivary calcium and sodium. PLoS One, 12:e0175437. 10.1371/journal.pone.0175437 PMC5396920 28422987

[57] Jackson CV, Holland AJ, Williams CA and Dickerson JW (1988). Vitamin E and Alzheimer's disease in subjects with Down's syndrome. J Ment Defic Res, 32(Pt 6):479-84. 10.1111/j.1365-2788.1988.tb01439.x 2976843

[58] Whittle N, Sartori SB, Dierssen M, Lubec G and Singewald N (2007). Fetal Down syndrome brains exhibit aberrant levels of neurotransmitters critical for normal brain development. Pediatrics, 120:e1465-71. 10.1542/peds.2006-3448 17998315

[59] Di Domenico F, Tramutola A and Butterfield DA (2017). Role of 4-hydroxy-2-nonenal (HNE) in the pathogenesis of alzheimer disease and other selected age-related neurodegenerative disorders. Free Radic Biol Med, 111:253-261. 10.1016/j.freeradbiomed.2016.10.490 27789292

[60] Busciglio J and Yankner BA (1995). Apoptosis and increased generation of reactive oxygen species in Down's syndrome neurons in vitro. Nature, 378:776-9. 10.1038/378776a0 8524410

[61] Di Domenico F, Coccia R, Cocciolo A, Murphy MP, Cenini G, Head E, et al. (2013). Impairment of proteostasis network in Down syndrome prior to the development of Alzheimer's disease neuropathology: redox proteomics analysis of human brain. Biochim Biophys Acta, 1832:1249-59. 10.1016/j.bbadis.2013.04.013 PMC3940071 23603808

[62] Necchi D, Pinto A, Tillhon M, Dutto I, Serafini MM, Lanni C, et al. (2015). Defective DNA repair and increased chromatin binding of DNA repair factors in Down syndrome fibroblasts. Mutat Res, 780:15-23. 10.1016/j.mrfmmm.2015.07.009 26258283

[63] Morawiec Z, Janik K, Kowalski M, Stetkiewicz T, Szaflik J, Morawiec-Bajda A, et al. (2008). DNA damage and repair in children with Down's syndrome. Mutat Res, 637:118-23. 10.1016/j.mrfmmm.2007.07.010 17765270

[64] Nunomura A, Perry G, Hirai K, Aliev G, Takeda A, Chiba S, et al. (1999). Neuronal RNA oxidation in Alzheimer's disease and Down's syndrome. Ann N Y Acad Sci, 893:362-4. 10.1111/j.1749-6632.1999.tb07855.x 10672267

[65] Perluigi M and Butterfield DA (2012). Oxidative Stress and Down Syndrome: A Route toward Alzheimer-Like Dementia. Curr Gerontol Geriatr Res, 2012:724904. 10.1155/2012/724904 PMC3235450 22203843

[66] Zana M, Szecsenyi A, Czibula A, Bjelik A, Juhasz A, Rimanoczy A, et al. (2006). Age-dependent oxidative stress-induced DNA damage in Down's lymphocytes. Biochem Biophys Res Commun, 345:726-33. 10.1016/j.bbrc.2006.04.167 16696946

[67] Zamponi E and Helguera PR (2019). The Shape of Mitochondrial Dysfunction in Down Syndrome. Dev Neurobiol, 79:613-621. 10.1002/dneu.22673 30830726

[68] Piccoli C, Izzo A, Scrima R, Bonfiglio F, Manco R, Negri R, et al. (2013). Chronic pro-oxidative state and mitochondrial dysfunctions are more pronounced in fibroblasts from Down syndrome foeti with congenital heart defects. Hum Mol Genet, 22:1218-32. 10.1093/hmg/dds529 23257287

[69] Singh D (2024). Revolutionizing cellular energy: The convergence of mitochondrial dynamics and delivery technologies. Mitochondrion, 76:101873. 10.1016/j.mito.2024.101873 38503363

[70] Izzo A, Nitti M, Mollo N, Paladino S, Procaccini C, Faicchia D, et al. (2017). Metformin restores the mitochondrial network and reverses mitochondrial dysfunction in Down syndrome cells. Hum Mol Genet, 26:1056-1069. 10.1093/hmg/ddx016 28087733

[71] Valenti D, Rossi L, Marzulli D, Bellomo F, De Rasmo D, Signorile A, et al. (2017). Inhibition of Drp1-mediated mitochondrial fission improves mitochondrial dynamics and bioenergetics stimulating neurogenesis in hippocampal progenitor cells from a Down syndrome mouse model. Biochim Biophys Acta Mol Basis Dis, 1863:3117-3127. 10.1016/j.bbadis.2017.09.014 28939434

[72] Wong H, Levenga J, Cain P, Rothermel B, Klann E and Hoeffer C (2015). RCAN1 overexpression promotes age-dependent mitochondrial dysregulation related to neurodegeneration in Alzheimer's disease. Acta Neuropathol, 130:829-43. 10.1007/s00401-015-1499-8 PMC4782929 26497675

[73] Predescu SA, Predescu DN, Knezevic I, Klein IK and Malik AB (2007). Intersectin-1s regulates the mitochondrial apoptotic pathway in endothelial cells. J Biol Chem, 282:17166-78. 10.1074/jbc.M608996200 17405881

[74] Iyer AM, van Scheppingen J, Milenkovic I, Anink JJ, Adle-Biassette H, Kovacs GG, et al. (2014). mTOR Hyperactivation in down syndrome hippocampus appears early during development. J Neuropathol Exp Neurol, 73:671-83. 10.1097/NEN.0000000000000083 24918639

[75] Wu W, Tian W, Hu Z, Chen G, Huang L, Li W, et al. (2014). ULK1 translocates to mitochondria and phosphorylates FUNDC1 to regulate mitophagy. EMBO Rep, 15:566-75. 10.1002/embr.201438501 PMC4210082 24671035

[76] Montgomery MK and Turner N (2015). Mitochondrial dysfunction and insulin resistance: an update. Endocr Connect, 4:R1-R15. 10.1530/EC-14-0092 PMC4261703 25385852

[77] Morton H, Kshirsagar S, Orlov E, Bunquin LE, Sawant N, Boleng L, et al. (2021). Defective mitophagy and synaptic degeneration in Alzheimer's disease: Focus on aging, mitochondria and synapse. Free Radic Biol Med, 172:652-667. 10.1016/j.freeradbiomed.2021.07.013 34246776

[78] Ganguly BB and Kadam NN (2022). Triplication of HSA21 on alterations in structure and function of mitochondria. Mitochondrion, 65:88-101. 10.1016/j.mito.2022.05.007 35623559

[79] Oliver DMA and Reddy PH (2019). Molecular Basis of Alzheimer's Disease: Focus on Mitochondria. J Alzheimers Dis, 72:S95-S116. 10.3233/JAD-190048 30932888

[80] Kim SH, Vlkolinsky R, Cairns N, Fountoulakis M and Lubec G (2001). The reduction of NADH ubiquinone oxidoreductase 24- and 75-kDa subunits in brains of patients with Down syndrome and Alzheimer's disease. Life Sci, 68:2741-50. 10.1016/s0024-3205(01)01074-8 11400916

[81] Valenti D, Tullo A, Caratozzolo MF, Merafina RS, Scartezzini P, Marra E, et al. (2010). Impairment of F1F0-ATPase, adenine nucleotide translocator and adenylate kinase causes mitochondrial energy deficit in human skin fibroblasts with chromosome 21 trisomy. Biochem J, 431:299-310. 10.1042/BJ20100581 20698827

[82] Murray A, Letourneau A, Canzonetta C, Stathaki E, Gimelli S, Sloan-Bena F, et al. (2015). Brief report: isogenic induced pluripotent stem cell lines from an adult with mosaic down syndrome model accelerated neuronal ageing and neurodegeneration. Stem Cells, 33:2077-84. 10.1002/stem.1968 PMC4737213 25694335

[83] Coskun PE, Wyrembak J, Derbereva O, Melkonian G, Doran E, Lott IT, et al. (2010). Systemic mitochondrial dysfunction and the etiology of Alzheimer's disease and down syndrome dementia. J Alzheimers Dis, 20 Suppl 2:S293-310. 10.3233/JAD-2010-100351 PMC4175722 20463402

[84] Liu Y, Borel C, Li L, Muller T, Williams EG, Germain PL, et al. (2017). Systematic proteome and proteostasis profiling in human Trisomy 21 fibroblast cells. Nat Commun, 8:1212. 10.1038/s41467-017-01422-6 PMC5663699 29089484

[85] Gleyzer N, Vercauteren K and Scarpulla RC (2005). Control of mitochondrial transcription specificity factors (TFB1M and TFB2M) by nuclear respiratory factors (NRF-1 and NRF-2) and PGC-1 family coactivators. Mol Cell Biol, 25:1354-66. 10.1128/MCB.25.4.1354-1366.2005 PMC548005 15684387

[86] Ventura-Clapier R, Garnier A and Veksler V (2008). Transcriptional control of mitochondrial biogenesis: the central role of PGC-1alpha. Cardiovasc Res, 79:208-17. 10.1093/cvr/cvn098 18430751

[87] Liu F, Liang Z, Wegiel J, Hwang YW, Iqbal K, Grundke-Iqbal I, et al. (2008). Overexpression of Dyrk1A contributes to neurofibrillary degeneration in Down syndrome. FASEB J, 22:3224-33. 10.1096/fj.07-104539 PMC2518253 18509201

[88] Lee S, Bang SM, Hong YK, Lee JH, Jeong H, Park SH, et al. (2016). The calcineurin inhibitor Sarah (Nebula) exacerbates Abeta42 phenotypes in a Drosophila model of Alzheimer's disease. Dis Model Mech, 9:295-306. 10.1242/dmm.018069 PMC4826976 26659252

[89] Ermak G, Sojitra S, Yin F, Cadenas E, Cuervo AM and Davies KJ (2012). Chronic expression of RCAN1-1L protein induces mitochondrial autophagy and metabolic shift from oxidative phosphorylation to glycolysis in neuronal cells. J Biol Chem, 287:14088-98. 10.1074/jbc.M111.305342 PMC3340208 22389495

[90] Izzo A, Manco R, Bonfiglio F, Cali G, De Cristofaro T, Patergnani S, et al. (2014). NRIP1/RIP140 siRNA-mediated attenuation counteracts mitochondrial dysfunction in Down syndrome. Hum Mol Genet, 23:4406-19. 10.1093/hmg/ddu157 24698981

[91] Rytinki MM and Palvimo JJ (2008). SUMOylation modulates the transcription repressor function of RIP140. J Biol Chem, 283:11586-95. 10.1074/jbc.M709359200 18211901

[92] Rytinki MM and Palvimo JJ (2009). SUMOylation attenuates the function of PGC-1alpha. J Biol Chem, 284:26184-93. 10.1074/jbc.M109.038943 PMC2758017 19625249

[93] Kanzleiter T, Rath M, Penkov D, Puchkov D, Schulz N, Blasi F, et al. (2014). Pknox1/Prep1 regulates mitochondrial oxidative phosphorylation components in skeletal muscle. Mol Cell Biol, 34:290-8. 10.1128/MCB.01232-13 PMC3911294 24216763

[94] Padilla J and Lee J (2021). A Novel Therapeutic Target, BACH1, Regulates Cancer Metabolism. Cells, 10. 10.3390/cells10030634 PMC8001775 33809182

[95] Bush A and Beail N (2004). Risk Factors for Dementia in People With Down Syndrome: Issues in Assessment and Diagnosis. American Journal on Mental Retardation, 109. 10.1352/0895-8017(2004)109<83:RFFDIP>2.0.CO;2 15000668

[96] Zana M, Janka Z and Kalman J (2007). Oxidative stress: a bridge between Down's syndrome and Alzheimer's disease. Neurobiol Aging, 28:648-76. 10.1016/j.neurobiolaging.2006.03.008 16624449

[97] Fortea J, Zaman SH, Hartley S, Rafii MS, Head E and Carmona-Iragui M (2021). Alzheimer's disease associated with Down syndrome: a genetic form of dementia. Lancet Neurol, 20:930-942. 10.1016/S1474-4422(21)00245-3 PMC9387748 34687637

[98] Lott IT and Head E (2019). Dementia in Down syndrome: unique insights for Alzheimer disease research. Nat Rev Neurol, 15:135-147. 10.1038/s41582-018-0132-6 PMC8061428 30733618

[99] Zhu X, Raina AK, Perry G and Smith MA (2004). Alzheimer's disease: the two-hit hypothesis. Lancet Neurol, 3:219-26. 10.1016/S1474-4422(04)00707-0 15039034

[100] Zhu X, Lee HG, Perry G and Smith MA (2007). Alzheimer disease, the two-hit hypothesis: an update. Biochim Biophys Acta, 1772:494-502. 10.1016/j.bbadis.2006.10.014 17142016

[101] Elangovan A, Babu HWS, Iyer M, Gopalakrishnan AV and Vellingiri B (2023). Untangle the mystery behind DS-associated AD - Is APP the main protagonist? Ageing Res Rev, 87:101930. 10.1016/j.arr.2023.101930 37031726

[102] Mucke L and Selkoe DJ (2012). Neurotoxicity of amyloid beta-protein: synaptic and network dysfunction. Cold Spring Harb Perspect Med, 2:a006338. 10.1101/cshperspect.a006338 PMC3385944 22762015

[103] John A and Reddy PH (2021). Synaptic basis of Alzheimer's disease: Focus on synaptic amyloid beta, P-tau and mitochondria. Ageing Res Rev, 65:101208. 10.1016/j.arr.2020.101208 PMC7770124 33157321

[104] Mori C, Spooner ET, Wisniewski KE, Wisniewski TM, Yamaguchi H, Saido TC, et al. (2009). Intraneuronal Aβ42 accumulation in Down syndrome brain. Amyloid, 9:88-102. 12440481

[105] Thirumalai S, Livesey FJ, Patani R and Hung C (2025). APP antisense oligonucleotides are effective in rescuing mitochondrial phenotypes in human iPSC-derived trisomy 21 astrocytes. Alzheimers Dement, 21:e14560. 10.1002/alz.14560 PMC11775556 39877983

[106] Ermak G, Pritchard MA, Dronjak S, Niu B and Davies KJ (2011). Do RCAN1 proteins link chronic stress with neurodegeneration? FASEB J, 25:3306-11. 10.1096/fj.11-185728 PMC3971512 21680892

[107] Lloret A, Badia MC, Giraldo E, Ermak G, Alonso MD, Pallardo FV, et al. (2011). Amyloid-beta toxicity and tau hyperphosphorylation are linked via RCAN1 in Alzheimer's disease. J Alzheimers Dis, 27:701-9. 10.3233/JAD-2011-110890 PMC3690537 21876249

[108] Royston MC, McKenzie JE, Gentleman SM, Sheng JG, Mann DM, Griffin WS, et al. (1999). Overexpression of s100beta in Down's syndrome: correlation with patient age and with beta-amyloid deposition. Neuropathol Appl Neurobiol, 25:387-93. 10.1046/j.1365-2990.1999.00196.x 10564528

[109] Ryoo SR, Jeong HK, Radnaabazar C, Yoo JJ, Cho HJ, Lee HW, et al. (2007). DYRK1A-mediated hyperphosphorylation of Tau. A functional link between Down syndrome and Alzheimer disease. J Biol Chem, 282:34850-7. 10.1074/jbc.M707358200 17906291

[110] Hayflick L and Moorhead PS (1961). The serial cultivation of human diploid cell strains. Exp Cell Res, 25:585-621. 10.1016/0014-4827(61)90192-6 13905658

[111] Munoz-Espin D and Serrano M (2014). Cellular senescence: from physiology to pathology. Nat Rev Mol Cell Biol, 15:482-96. 10.1038/nrm3823 24954210

[112] Lopez-Otin C, Blasco MA, Partridge L, Serrano M and Kroemer G (2013). The hallmarks of aging. Cell, 153:1194-217. 10.1016/j.cell.2013.05.039 PMC3836174 23746838

[113] Jurk D, Wang C, Miwa S, Maddick M, Korolchuk V, Tsolou A, et al. (2012). Postmitotic neurons develop a p21-dependent senescence-like phenotype driven by a DNA damage response. Aging Cell, 11:996-1004. 10.1111/j.1474-9726.2012.00870.x PMC3533793 22882466

[114] Musi N, Valentine JM, Sickora KR, Baeuerle E, Thompson CS, Shen Q, et al. (2018). Tau protein aggregation is associated with cellular senescence in the brain. Aging Cell, 17:e12840. 10.1111/acel.12840 PMC6260915 30126037

[115] Kalanj-Bognar S, Rundek T, Furac I, Demarin V and Cosovic C (2002). Leukocyte lysosomal enzymes in Alzheimer's disease and Down's syndrome. J Gerontol A Biol Sci Med Sci, 57:B16-21. 10.1093/gerona/57.1.b16 11773202

[116] Rueda N, Vidal V, Garcia-Cerro S, Narcis JO, Llorens-Martin M, Corrales A, et al. (2018). Anti-IL17 treatment ameliorates Down syndrome phenotypes in mice. Brain Behav Immun, 73:235-251. 10.1016/j.bbi.2018.05.008 29758264

[117] Ahmed AA, Smoczer C, Pace B, Patterson D and Cress Cabelof D (2018). Loss of DNA polymerase beta induces cellular senescence. Environ Mol Mutagen, 59:603-612. 10.1002/em.22206 PMC6203593 29968395

[118] Meharena HS, Marco A, Dileep V, Lockshin ER, Akatsu GY, Mullahoo J, et al. (2022). Down-syndrome-induced senescence disrupts the nuclear architecture of neural progenitors. Cell Stem Cell, 29:116-130 e7. 10.1016/j.stem.2021.12.002 PMC8805993 34995493

[119] Adorno M, Sikandar S, Mitra SS, Kuo A, Nicolis Di Robilant B, Haro-Acosta V, et al. (2013). Usp16 contributes to somatic stem-cell defects in Down's syndrome. Nature, 501:380-4. 10.1038/nature12530 PMC3816928 24025767

[120] Pawlikowski B, Betta ND, Elston T, Williams DA and Olwin BB (2018). Muscle stem cell dysfunction impairs muscle regeneration in a mouse model of Down syndrome. Sci Rep, 8:4309. 10.1038/s41598-018-22342-5 PMC5844921 29523805

[121] Contestabile A, Fila T, Ceccarelli C, Bonasoni P, Bonapace L, Santini D, et al. (2007). Cell cycle alteration and decreased cell proliferation in the hippocampal dentate gyrus and in the neocortical germinal matrix of fetuses with Down syndrome and in Ts65Dn mice. Hippocampus, 17:665-78. 10.1002/hipo.20308 17546680

[122] Sukenik-Halevy R, Biron-Shental T, Sharony R, Fejgin MD and Amiel A (2011). Telomeres in trisomy 21 amniocytes. Cytogenet Genome Res, 135:12-8. 10.1159/000329714 21734364

[123] Amiel A, Fejgin MD, Liberman M, Sharon Y, Kidron D and Biron-Shental T (2013). Senescence in amniocytes and placentas from trisomy 21 pregnancies. J Matern Fetal Neonatal Med, 26:1086-9. 10.3109/14767058.2013.768982 23339291

[124] Rodriguez-Sureda V, Vilches A, Sanchez O, Audi L and Dominguez C (2015). Intracellular oxidant activity, antioxidant enzyme defense system, and cell senescence in fibroblasts with trisomy 21. Oxid Med Cell Longev, 2015:509241. 10.1155/2015/509241 PMC4380103 25852816

[125] Lockrow J, Prakasam A, Huang P, Bimonte-Nelson H, Sambamurti K and Granholm AC (2009). Cholinergic degeneration and memory loss delayed by vitamin E in a Down syndrome mouse model. Exp Neurol, 216:278-89. 10.1016/j.expneurol.2008.11.021 PMC2704550 19135442

[126] Sano M, Aisen PS, Andrews HF, Tsai WY, Lai F, Dalton AJ, et al. (2016). Vitamin E in aging persons with Down syndrome: A randomized, placebo-controlled clinical trial. Neurology, 86:2071-6. 10.1212/WNL.0000000000002714 PMC4891209 27164691

[127] Lee P and Ulatowski LM (2019). Vitamin E: Mechanism of transport and regulation in the CNS. IUBMB Life, 71:424-429. 10.1002/iub.1993 30556640

[128] Parisotto EB, Giaretta AG, Zamoner A, Moreira EA, Frode TS, Pedrosa RC, et al. (2015). Persistence of the benefit of an antioxidant therapy in children and teenagers with Down syndrome. Res Dev Disabil, 45-46:14-20. 10.1016/j.ridd.2015.07.010 26207872

[129] Parisotto EB, Garlet TR, Cavalli VL, Zamoner A, da Rosa JS, Bastos J, et al. (2014). Antioxidant intervention attenuates oxidative stress in children and teenagers with Down syndrome. Res Dev Disabil, 35:1228-36. 10.1016/j.ridd.2014.03.013 24685938

[130] Tiano L, Padella L, Santoro L, Carnevali P, Principi F, Bruge F, et al. (2012). Prolonged coenzyme Q10 treatment in Down syndrome patients: effect on DNA oxidation. Neurobiol Aging, 33:626 e1-8. 10.1016/j.neurobiolaging.2011.03.025 21601315

[131] Larsen EL, Padella L, Bergholdt HKM, Henriksen T, Santoro L, Gabrielli O, et al. (2018). The effect of long-term treatment with coenzyme Q10 on nucleic acid modifications by oxidation in children with Down syndrome. Neurobiol Aging, 67:159-161. 10.1016/j.neurobiolaging.2018.03.001 29665577

[132] Santos RA, Costa LH, Linhares RC, Pradella-Hallinan M, Coelho FMS and Oliveira GDP (2022). Sleep disorders in Down syndrome: a systematic review. Arq Neuropsiquiatr, 80:424-443. 10.1590/0004-282X-ANP-2021-0242 PMC9173224 35293557

[133] Uberos J, Romero J, Molina-Carballo A and Munoz-Hoyos A (2010). Melatonin and elimination of kynurenines in children with Down's syndrome. J Pediatr Endocrinol Metab, 23:277-82. 10.1515/jpem.2010.23.3.277 20480727

[134] Parisotto EB, Vidal V, Garcia-Cerro S, Lantigua S, Wilhelm Filho D, Sanchez-Barcelo EJ, et al. (2016). Chronic Melatonin Administration Reduced Oxidative Damage and Cellular Senescence in the Hippocampus of a Mouse Model of Down Syndrome. Neurochem Res, 41:2904-2913. 10.1007/s11064-016-2008-8 27450081

[135] Corrales A, Martinez P, Garcia S, Vidal V, Garcia E, Florez J, et al. (2013). Long-term oral administration of melatonin improves spatial learning and memory and protects against cholinergic degeneration in middle-aged Ts65Dn mice, a model of Down syndrome. J Pineal Res, 54:346-58. 10.1111/jpi.12037 23350971

[136] Vacca RA, Valenti D, Caccamese S, Daglia M, Braidy N and Nabavi SM (2016). Plant polyphenols as natural drugs for the management of Down syndrome and related disorders. Neurosci Biobehav Rev, 71:865-877. 10.1016/j.neubiorev.2016.10.023 27826066

[137] Schroeder EK, Kelsey NA, Doyle J, Breed E, Bouchard RJ, Loucks FA, et al. (2009). Green tea epigallocatechin 3-gallate accumulates in mitochondria and displays a selective antiapoptotic effect against inducers of mitochondrial oxidative stress in neurons. Antioxid Redox Signal, 11:469-80. 10.1089/ars.2008.2215 PMC13148728 18754708

[138] Valenti D, De Rasmo D, Signorile A, Rossi L, de Bari L, Scala I, et al. (2013). Epigallocatechin-3-gallate prevents oxidative phosphorylation deficit and promotes mitochondrial biogenesis in human cells from subjects with Down's syndrome. Biochim Biophys Acta, 1832:542-52. 10.1016/j.bbadis.2012.12.011 23291000

[139] de la Torre R, de Sola S, Hernandez G, Farre M, Pujol J, Rodriguez J, et al. (2016). Safety and efficacy of cognitive training plus epigallocatechin-3-gallate in young adults with Down's syndrome (TESDAD): a double-blind, randomised, placebo-controlled, phase 2 trial. Lancet Neurol, 15:801-810. 10.1016/S1474-4422(16)30034-5 27302362

[140] Valenti D, de Bari L, de Rasmo D, Signorile A, Henrion-Caude A, Contestabile A, et al. (2016). The polyphenols resveratrol and epigallocatechin-3-gallate restore the severe impairment of mitochondria in hippocampal progenitor cells from a Down syndrome mouse model. Biochim Biophys Acta, 1862:1093-104. 10.1016/j.bbadis.2016.03.003 26964795

[141] Brasnyo P, Molnar GA, Mohas M, Marko L, Laczy B, Cseh J, et al. (2011). Resveratrol improves insulin sensitivity, reduces oxidative stress and activates the Akt pathway in type 2 diabetic patients. Br J Nutr, 106:383-9. 10.1017/S0007114511000316 21385509

[142] Mollo N, Cicatiello R, Aurilia M, Scognamiglio R, Genesio R, Charalambous M, et al. (2020). Targeting Mitochondrial Network Architecture in Down Syndrome and Aging. Int J Mol Sci, 21. 10.3390/ijms21093134 PMC7247689 32365535

[143] Wang X, Su B, Lee HG, Li X, Perry G, Smith MA, et al. (2009). Impaired balance of mitochondrial fission and fusion in Alzheimer's disease. J Neurosci, 29:9090-103. 10.1523/JNEUROSCI.1357-09.2009 PMC2735241 19605646

[144] Hall AR, Burke N, Dongworth RK and Hausenloy DJ (2014). Mitochondrial fusion and fission proteins: novel therapeutic targets for combating cardiovascular disease. Br J Pharmacol, 171:1890-906. 10.1111/bph.12516 PMC3976611 24328763

[145] Mollo N, Nitti M, Zerillo L, Faicchia D, Micillo T, Accarino R, et al. (2019). Pioglitazone Improves Mitochondrial Organization and Bioenergetics in Down Syndrome Cells. Front Genet, 10:606. 10.3389/fgene.2019.00606 PMC6609571 31316549

[146] Chen Y, Chen J, Sun X, Shi X, Wang L, Huang L, et al. (2018). Evaluation of the neuroprotective effect of EGCG: a potential mechanism of mitochondrial dysfunction and mitochondrial dynamics after subarachnoid hemorrhage. Food Funct, 9:6349-6359. 10.1039/c8fo01497c 30452052

[147] Lamming DW (2016). Inhibition of the Mechanistic Target of Rapamycin (mTOR)-Rapamycin and Beyond. Cold Spring Harb Perspect Med, 6. 10.1101/cshperspect.a025924 PMC4852795 27048303

[148] Di Domenico F, Tramutola A, Barone E, Lanzillotta C, Defever O, Arena A, et al. (2019). Restoration of aberrant mTOR signaling by intranasal rapamycin reduces oxidative damage: Focus on HNE-modified proteins in a mouse model of down syndrome. Redox Biol, 23:101162. 10.1016/j.redox.2019.101162 PMC6859577 30876754

[149] Pradeepkiran JA and Reddy PH (2020). Defective mitophagy in Alzheimer's disease. Ageing Res Rev, 64:101191. 10.1016/j.arr.2020.101191 PMC7710581 33022416

[150] Pradeepkiran JA, Baig J, Seman A and Reddy PH (2024). Mitochondria in Aging and Alzheimer's Disease: Focus on Mitophagy. Neuroscientist, 30:440-457. 10.1177/10738584221139761 36597577

[151] Andreux PA, Blanco-Bose W, Ryu D, Burdet F, Ibberson M, Aebischer P, et al. (2019). The mitophagy activator urolithin A is safe and induces a molecular signature of improved mitochondrial and cellular health in humans. Nat Metab, 1:595-603. 10.1038/s42255-019-0073-4 32694802

[152] Tran M and Reddy PH (2020). Defective Autophagy and Mitophagy in Aging and Alzheimer's Disease. Front Neurosci, 14:612757. 10.3389/fnins.2020.612757 PMC7820371 33488352

[153] Kirkland JL and Tchkonia T (2020). Senolytic drugs: from discovery to translation. J Intern Med, 288:518-536. 10.1111/joim.13141 PMC7405395 32686219

[154] Roberts AW, Davids MS, Pagel JM, Kahl BS, Puvvada SD, Gerecitano JF, et al. (2016). Targeting BCL2 with Venetoclax in Relapsed Chronic Lymphocytic Leukemia. N Engl J Med, 374:311-22. 10.1056/NEJMoa1513257 PMC7107002 26639348

[155] Vacurova E, Vlachova E, Stursa J, Bohacova K, Havrlantova T, Skop V, et al. (2025). Targeting Mitochondrial Integrity as a New Senolytic Strategy. Aging Dis. 10.14336/AD.2024.1100 PMC12539531 39965253

[156] Iulita MF, Bejanin A, Vilaplana E, Carmona-Iragui M, Benejam B, Videla L, et al. (2023). Association of biological sex with clinical outcomes and biomarkers of Alzheimer's disease in adults with Down syndrome. Brain Commun, 5:fcad074. 10.1093/braincomms/fcad074 PMC10088472 37056479

[157] Del Hoyo Soriano L, Wagemann O, Bejanin A, Levin J and Fortea J (2025). Sex-related differences in genetically determined Alzheimer's disease. Front Aging Neurosci, 17:1522434. 10.3389/fnagi.2025.1522434 PMC11913828 40103931

[158] Zhu X, Lee HG, Casadesus G, Avila J, Drew K, Perry G, et al. (2005). Oxidative imbalance in Alzheimer's disease. Mol Neurobiol, 31:205-17. 10.1385/MN:31:1-3:205 15953822

[159] Ludtmann MHR and Abramov AY (2018). Mitochondrial calcium imbalance in Parkinson's disease. Neurosci Lett, 663:86-90. 10.1016/j.neulet.2017.08.044 28838811

[160] D'Egidio F, Castelli V, Cimini A and d'Angelo M (2023). Cell Rearrangement and Oxidant/Antioxidant Imbalance in Huntington's Disease. Antioxidants (Basel), 12. 10.3390/antiox12030571 PMC10045781 36978821

[161] Zuo X, Zhou J, Li Y, Wu K, Chen Z, Luo Z, et al. (2021). TDP-43 aggregation induced by oxidative stress causes global mitochondrial imbalance in ALS. Nat Struct Mol Biol, 28:132-142. 10.1038/s41594-020-00537-7 33398173

